# Biological Performance and Molecular Mechanisms of Mesyl MicroRNA-Targeted Oligonucleotides in Colorectal Cancer Cells

**DOI:** 10.3390/ijms262311747

**Published:** 2025-12-04

**Authors:** Svetlana K. Miroshnichenko, Olga A. Patutina, Andrey V. Markov, Maxim S. Kupryushkin, Valentin V. Vlassov, Marina A. Zenkova

**Affiliations:** Institute of Chemical Biology and Fundamental Medicine Siberian Branch of the Russian Academy of Sciences, 630090 Novosibirsk, Russia; sveta-mira@yandex.ru (S.K.M.); patutina@1bio.ru (O.A.P.); andmrkv@gmail.com (A.V.M.); kuprummax@1bio.ru (M.S.K.); vvv@niboch.nsc.ru (V.V.V.)

**Keywords:** colorectal adenocarcinoma, antisense oligonucleotides, antimiRs, microRNA, miR-21, miR-17, miR-155, proteomic profiling, mesyl oligonucleotides

## Abstract

Colorectal cancer (CRC) remains one of the most aggressive and therapeutically resistant malignancies worldwide. This study examined the molecular mechanisms underlying the anti-oncogenic activity of methanesulfonyl phosphoramidate-modified antisense oligonucleotides (µ-ASOs) targeting miR-21, miR-17, and miR-155, which represent critical oncogenic drivers in CRC. Using human colorectal adenocarcinoma Caco-2 cells transfected with either individual µ-ASOs or their triple combination, we assessed target miRNA downregulation, antiproliferative and anti-migratory activities, and performed extensive proteomic profiling. Protein–protein interaction network analysis of differentially expressed proteins (DEPs) revealed that, beyond modulation of core metabolic processes, each µ-ASO exhibited distinct effects: µ-21 predominantly affected apoptosis, cell cycle, and DNA repair; µ-17 influenced proliferation and chaperone responses; and µ-155 modulated intracellular transport and immune regulation. Combination treatment elicited a unique proteomic signature partially overlapping with monotherapies. The proteomic analysis revealed several validated and putative miRNA-targeted DEPs, including both established and novel candidates in the CRC context: RPL31, CCT2, and CDC37 (miR-21); DNM2, SNRPN, NUP98, and NUP85 (miR-17); as well as RPL17 (miR-155). Expression of these targets correlated with favorable clinical outcomes in CRC patients. This work provides the first comprehensive mechanistic insight into antisense oligonucleotide-mediated miRNA suppression in Caco-2 colorectal adenocarcinoma cells and expands the miRNA target landscape.

## 1. Introduction

Colorectal cancer (CRC) remains a highly aggressive and therapeutically challenging malignancy, accounting for a significant proportion of cancer-related mortality worldwide [1]. The lack of reliable early diagnostic tools severely limits the efficacy of therapeutic interventions, while conventional chemotherapy regimens are often hampered by systemic toxicity and poor patient response. Consequently, the development of specific, low-toxic, and highly effective agents targeting key molecular drivers of CRC represents a critical unmet need in modern molecular oncology.

Among the promising therapeutic targets are short non-coding RNAs, particularly miRNAs. These molecules play a pivotal role in cellular homeostasis due to their multifunctional, multitarget nature, influencing virtually all aspects of cell physiology [2,3]. Dysregulation of miRNA expression and function is increasingly recognized as a fundamental driver of cancer pathogenesis, directly contributing to the acquisition of malignant traits, including uncontrolled proliferation, genomic instability, immune evasion, increased metastatic potential, resistance to apoptosis, cell cycle dysregulation, etc. [4].

Of particular significance to CRC development are three miRNAs: miR-17, miR-21, and miR-155. Overexpression of these miRNAs in colorectal tumors has been shown to be strongly correlated with advanced pathological stages and disease progression, underscoring their potential as both diagnostic markers and therapeutic targets [5]. Current evidence indicates that these miRNAs exert their effects through broad signaling cascades, enabling comprehensive modulation of cellular functions.

miR-21 is one of the most extensively studied miRNAs in CRC pathogenesis. Accumulating clinical evidence demonstrates that elevated miR-21 expression in tumor tissues strongly correlates with poor patient survival and advanced tumor node metastasis (TNM) staging [6]. Mechanistically, miR-21 exerts its oncogenic effects through multiple critical pathways. For instance, miR-21 suppresses PTEN to hyperactivate Akt-mediated proliferation and autophagy, promotes angiogenesis via the VEGF signaling cascade, and exacerbates tumor-associated inflammation through NF-κB signaling [7,8,9]. It also orchestrates chemoresistance by inhibiting the tumor suppressor PDCD4, thereby unleashing JNK pathway activity [10]. Furthermore, this miRNA amplifies malignant transformation by targeting RASA1, leading to dysregulated RAS signaling and enhanced aggressiveness [11].

Functional analysis has demonstrated that miR-17 primarily regulates CRC progression via the TGF-β signaling pathway [12]. It was shown that among all miRNAs studied in the CRC context, miR-17 possessed the highest number of direct targets within this cascade [12]. Moreover, it was demonstrated that miR-17 drives CRC aggressiveness via a self-reinforcing cancer-associated fibroblast (CAF)-tumor loop: exosomal miR-17 activates RUNX3/MYC/TGF-β1 signaling, hyperactivating CAFs to enhance oncogenic signaling and promote metastasis [13]. The next important cascade for miR-17 is WNT/β-catenin signaling, where the key regulated target for miR-17 is the tumor suppressor protein P130 [14]. Among its individual targets in CRC, CADM2 and HSBP2 have been identified as critical mediators of miR-17 oncogenic effects [15,16]. CADM2 suppression by miR-17 promotes tumor cell proliferation, migration, invasion, and dysregulation of the cell cycle [16], while HSBP2 downregulation disrupts programmed cell death, enhances clonogenic potential, and increases tumor cell survival [15].

Among the three studied miRNAs, miR-155 displays the most specialized functional profile, with current evidence predominantly linking its dysregulation with the promotion of metastatic processes in CRC [17]. Its pro-metastatic effects are largely mediated through activation of the JAK2/STAT3/NF-κB signaling axis [17]. Additionally, miR-155 modulates colorectal cancer cell motility by targeting components of the WNT/β-catenin pathway [18,19], which simultaneously regulates tumor cell proliferation and invasion [19]. Beyond canonical signaling pathways, miR-155 coordinates migration, invasion, and cell cycle progression through direct regulation of specific molecular targets, including FOXO3a and RhoA [20,21].

Given the pivotal roles of these oncogenic miRNAs in driving CRC progression and malignancy, targeted suppression of their expression and activity using sequence-specific oligonucleotide therapeutics may offer a comprehensive strategy to disrupt multiple carcinogenic pathways simultaneously.

Our research group has recently characterized the properties and biological activity of miRNA-targeted antisense oligonucleotides incorporating a newly developed methanesulfonyl phosphoramidate (µ-) modification of internucleotidic bonds (µ-ASOs) [22,23]. We identified several advantageous characteristics of µ-ASOs, including exceptional nuclease resistance, RNase H-activating capability, and low toxicity [22]. Along with this, we demonstrated that paired combinations of µ-ASOs, targeting miR-21, miR-17, and miR-155, synergistically suppressed proliferation and migration of murine B16 melanoma cells in vitro, while the triple combination of µ-ASOs exhibited highly potent inhibition of metastasis in the B16 model, as well as significant suppression of tumor growth in murine RLS_40_ lymphosarcoma in vivo, resulting in near-complete inhibition of tumor cell mitotic activity [24].

In the present study, we extended our investigation of miRNA-targeted µ-ASO therapy to a histopathologically and molecularly distinct cancer model, specifically epithelial colorectal carcinoma. Unlike murine melanoma and lymphosarcoma, which represent mesenchymal and hematopoietic malignancies, colorectal cancer is an epithelial-derived adenocarcinoma with distinct genetic alterations, signaling pathways, and therapeutic responses. For this purpose, we selected a clinically relevant model: human colorectal adenocarcinoma Caco-2 cells. Notably, the present study advances beyond predominantly phenotypic studies by incorporating systematic proteomic profiling. This approach enables comprehensive characterization of the molecular mechanisms underlying µ-ASO therapeutic efficacy, revealing how miRNA suppression translates into proteomic alterations and identifying potential molecular determinants of treatment response. Based on the complementary roles of miR-21, miR-17, and miR-155 in CRC pathogenesis and the synergistic effects of paired µ-ASO combinations observed in our previous work [24], we hypothesized that simultaneous suppression of these three miRNAs would produce therapeutically significant molecular alterations in CRC cells through coordinated disruption of specifically distinct and interconnected oncogenic pathways. We therefore evaluated individual µ-ASOs and their triple combination to determine whether coordinated targeting generates integrated or unique proteomic responses. Thus, this study aimed to (1) characterize proteomic alterations induced by individual µ-ASOs targeting miR-21, miR-17, or miR-155 in Caco-2 cells to identify miRNA-specific molecular signatures, (2) analyze the combinatorial effects of the triple µ-ASO combination at the proteome level compared to individual treatments, and (3) elucidate the molecular mechanisms underlying µ-ASO therapeutic efficacy in human colorectal cancer.

## 2. Results

### 2.1. Biological Performance of µ-ASO in Caco-2 Cells

Biological performance of miRNA-targeted oligonucleotides was assessed by their capacity to modulate tumor cell viability, proliferation, and migration. The cells were transfected with µ-ASOs in complex with Lipofectamine 2000^TM^, which provided high efficiency of oligonucleotide delivery to Caco-2 cells (Figure S1).

**Figure 1:**
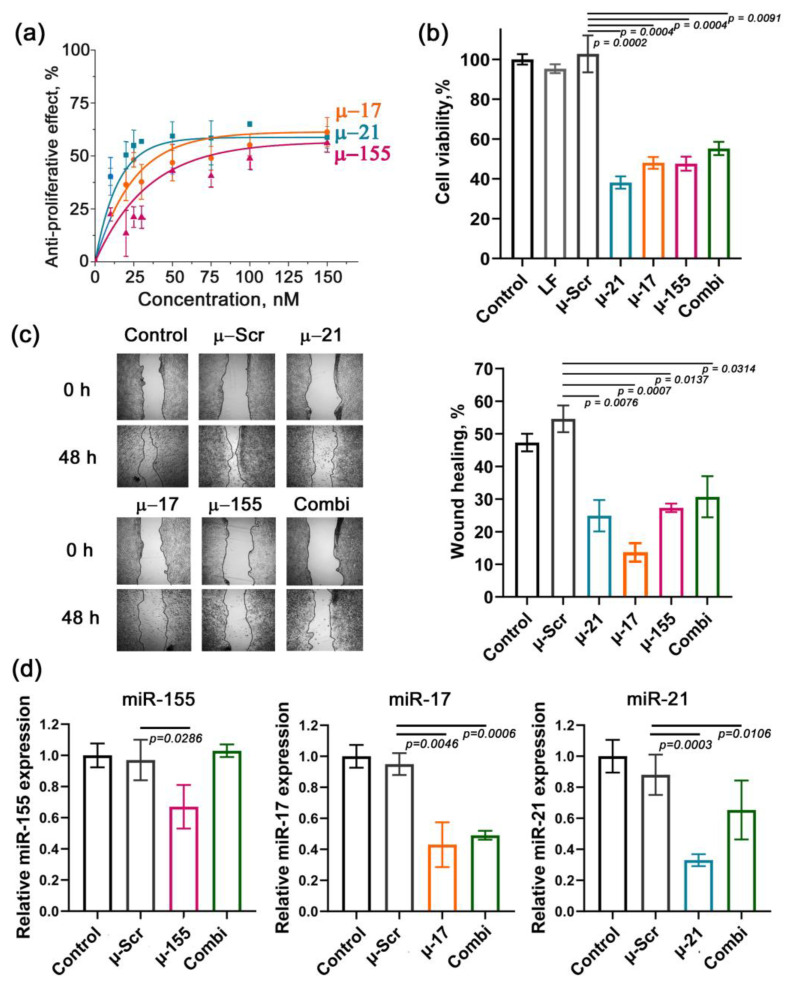
Biological performance of µ-ASOs in Caco-2 cells. (**a**) Dose-dependent anti-proliferative effects of µ-21, µ-17, and µ-155 oligonucleotides (10–150 nM) 72 h post-transfection with µ-ASOs. (**b**) Viability of Caco-2 cells 72 h following cell transfection with a single µ-ASO (120 nM) or triple combination of µ-ASOs (40 nM each). (**c**) Anti-migratory effect of a single µ-ASO (120 nM) or triple combination of µ-ASOs (40 nM each) assessed by wound-healing assay. Representative images (left panel) show scratch boundaries (black lines) at 0 h and 48 h post-scratching (4× magnification). In the right panel, the degree of wound healing after 48 h is shown. (**d**) RT-qPCR data showing miRNA expression levels 48 h after cell transfection with a single µ-ASO (120 nM) or triple combination of µ-ASOs (40 nM each). miR-21, miR-17, and miR-155 levels were normalized to U6 snRNA expression. Control—cells without treatment; LF—cells incubated with Lipofectamine 2000^TM^; µ-Scr, µ-21, µ-17, and µ-155—cells transfected with scrambled or miRNA-targeted µ-ASO in complex with Lipofectamine 2000^TM^, Combi—cells transfected with a triple combination of µ-ASOs in complex with Lipofectamine 2000^TM^ (40 nM each, 120 nM total). Data represent mean ± SEM of ≥3 independent experiments. Significance was determined by one-way ANOVA with the post hoc Tukey test (*p*-values represent significant differences from µ-Scr).

Evaluation of µ-ASO effects on tumor cell survival and proliferation revealed dose-dependent antiproliferative effects in Caco-2 cells (Figure 1a). Comparative analysis of concentration–response relationships demonstrated that a 50% reduction in tumor cell proliferation was achieved at concentrations above 75 nM for µ-155, whereas µ-17 required concentrations exceeding 35 nM (Figure 1a). Notably, µ-21 exhibited the most potent antiproliferative activity, suppressing tumor cell growth by 50% at 20 nM (Figure 1a). At the maximal tested concentration (150 nM), all µ-ASOs showed comparable efficacy, with 55–65% inhibition of cell proliferation (Figure 1a). In this study, we also assessed the potential of a combination of all three µ-ASOs (µ-21 + µ-17 + µ-155, hereafter termed Combi). Based on dose–response analysis of Caco-2 cell viability, we determined that the optimal concentration for each µ-ASO in Combi is 40 nM, since the inhibitory effect of individual oligonucleotides plateaus at this dose, achieving approximately 50% suppression. Thus, the resulting concentration of each oligonucleotide applied individually or in Combi was 120 nM. At this concentration, single µ-ASOs exhibit a 52% (µ-17, µ-155) and 60% (µ-21) decrease in Caco-2 cell viability, while transfection of Combi resulted in 45% inhibition of tumor cell viability (Figure 1b).

Analysis of µ-ASO-mediated modulation of Caco-2 cell migration using wound-healing assays demonstrated that µ-21, µ-155, and Combi caused comparable suppression of migration, leading to 30, 27, and 24% reduction, respectively, compared to the scrambled control (µ-Scr) (Figure 1c). Notably, µ-17 demonstrated the most potent antimigratory effect, achieving 41% inhibition of Caco-2 cell migration relative to µ-Scr (Figure 1c).

Evaluation of target miRNA suppression showed that 48 h post-transfection, the least pronounced reduction in miRNA level (35%) was observed for miR-155 (Figure 1d). In contrast, µ-17 demonstrated robust 57% suppression of miR-17 (Figure 1d). The most substantial and statistically significant decrease, up to 67%, was observed for miR-21 upon µ-21 treatment (Figure 1d). It should be mentioned that Combi, which has a threefold lower concentration of each µ-ASO (40 nM each), lacks a significant inhibitory effect in terms of miR-155, but results in 35% and 50% downregulation of miR-17 and miR-21, respectively (Figure 1d).

In summary, our findings demonstrate distinct biological profiles among the tested µ-ASOs: µ-155 exhibits moderate suppression of its target miRNA; however, there is a significant reduction in Caco-2 cell migration and viability. µ-21 and µ-17 combine significant downregulation of miR-21 and miR-17, respectively, robust antiproliferative effects, and pronounced anti-migratory activity. Notably, the triple combination (Combi) showed differential yet significant suppression of both miR-21 and miR-17 in tumor cells but exerted substantial antiproliferative and anti-migrative activity.

### 2.2. Proteomic Profiling of Caco-2 Cells Following Treatment with µ-ASOs Targeted to miR-21, miR-17, and miR-155

To unveil the molecular underpinnings of µ-ASOs’ action, we conducted a proteomic profiling analysis of Caco-2 cells 72 h post-transfection with individual µ-ASOs or Combi (total concentration was 120 nM, pre-complexed with Lipofectamine 2000™). This time point was strategically chosen based on the kinetics of µ-ASO-mediated miRNA suppression, where maximum inhibition is achieved at 48 h (Figure S2). Given the inherent inertia of the miRNA-mediated regulatory response, we hypothesized that consequent proteome-wide alterations would be most reliably detected 24 h after this peak of miRNA knockdown.

For proteomic analysis, the following groups of Caco-2 cells were used: Control—intact Caco-2; µ-Scr—Caco-2 cells transfected with control oligonucleotide that has no homology to mammalian genomes (120 nM); µ-21, µ-17, and µ-155—Caco-2 cells treated with oligonucleotides targeting miR-21, miR-17, and miR-155, respectively (120 nM); Combi—Caco-2 cells treated with a combination of three oligonucleotides (µ-21, µ-17, and µ-155) at 40 nM each (total concentration 120 nM). Transfection of all µ-ASOs oligonucleotides was conducted in a total concentration of 120 nM pre-complexed with Lipofectamine 2000™. 72 h after transfection, cell samples were lysed, and 100 µg of total protein was analyzed by LC–MS/MS.

**Figure 2:**
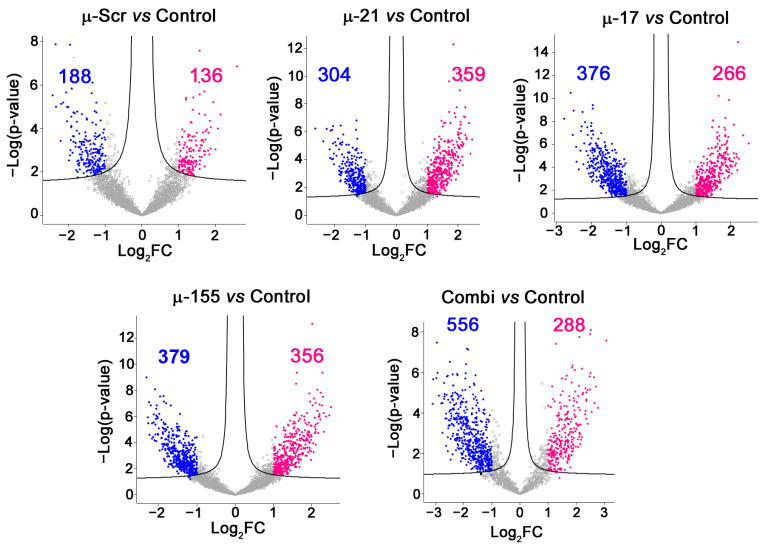
Proteomic alterations upon µ-ASO treatment of Caco-2 cells. Volcano plots showing the number of differentially expressed proteins (DEPs) in Caco-2 cells 72 h following transfection with control oligonucleotide (µ-Scr), miRNA-targeted µ-21, µ-17, and µ-155 or their combination Combi (µ-21 + µ-17 + µ-155) pre-complexed with Lipofectamine 2000^TM^ (total concentration 120 nM) relative to Control (intact Caco-2 cells). Proteins with |log_2_(Fold Change (FC))| ≥1 (adj. *p*-value ˂ 0.05) are highlighted in pink (upregulated) and blue (downregulated). Gray dots represent non-significant changes. Analysis was performed using Perseus (v 2.0.11).

LC–MS/MS analysis of proteins isolated from µ-ASO-treated and control Caco-2 cells after stringent contaminant filtering identified 3,957 high-confidence proteins (≥2 unique peptides/protein). Pairwise comparisons against untreated control revealed differentially expressed proteins (DEPs) with ≥2-fold change (log_2_FC ≥1, *p*-value ˂ 0.05), including µ-Scr—324 DEPs; µ-21—663 DEPs; µ-17—642 DEPs; µ-155—735 DEPs, and Combi—844 DEPs (Figure 2). While µ-Scr, µ-21, and µ-155-treated cells exhibited near-balanced up-/downregulation, µ-17 and Combi cohorts exhibited a consistent downregulation trend: 376 and 556 downregulated proteins vs. 266 and 288 upregulated proteins for µ-17 and Combi, respectively (Figure 2).

Principal component analysis revealed clear separation of groups treated with µ-17, µ-155, and Combi along PC1 from intact Caco-2 cells (control) or groups transfected with control µ-Scr oligonucleotide and µ-21 (Figure S3a). Pronounced segregation of µ-21 and µ-155-treated samples from the Combi cohort was found along PC2, suggesting distinct mechanisms of action between individual oligonucleotides and their combination (Figure S3a). Hierarchical clustering of proteomic profiles revealed compound-dependent stratification, additionally confirming differences in the activity of µ-17, µ-155, Combi, and µ-21 groups compared with µ-Scr and Control, as well as the reproducibility of results within each group (Figure S3b).

#### 2.2.1. Major Common Biological Processes and Signaling Pathways Regulated by µ-ASOs, Targeting miR-21, miR-17, and miR-155

The obtained proteomic datasets were subjected to systematic bioinformatics analysis to identify the most differentially expressed protein targets and functionally enriched pathways mediated by µ-ASO treatment. At the first step of the study, we compared the complete sets of DEPs exhibiting at least a twofold change obtained for the control oligonucleotide (µ-Scr) and each of the single miRNA-targeted µ-ASOs (µ-21, µ-17, or µ-155). This analysis was performed to identify potential overlapping proteins shared by all four groups, which could indicate a direct influence of the mesyl modification on the Caco-2 cell proteome. As a result, the intersection of the corresponding four sets of DEPs revealed no overlapping proteins common to all groups (Figure S4). The absence of shared DEPs suggests that the µ-modification itself does not induce nonspecific cytotoxic effects in this cell type. Further study is focused specifically on elucidating the effects of specific miRNA-targeted µ-ASOs.

**Figure 3:**
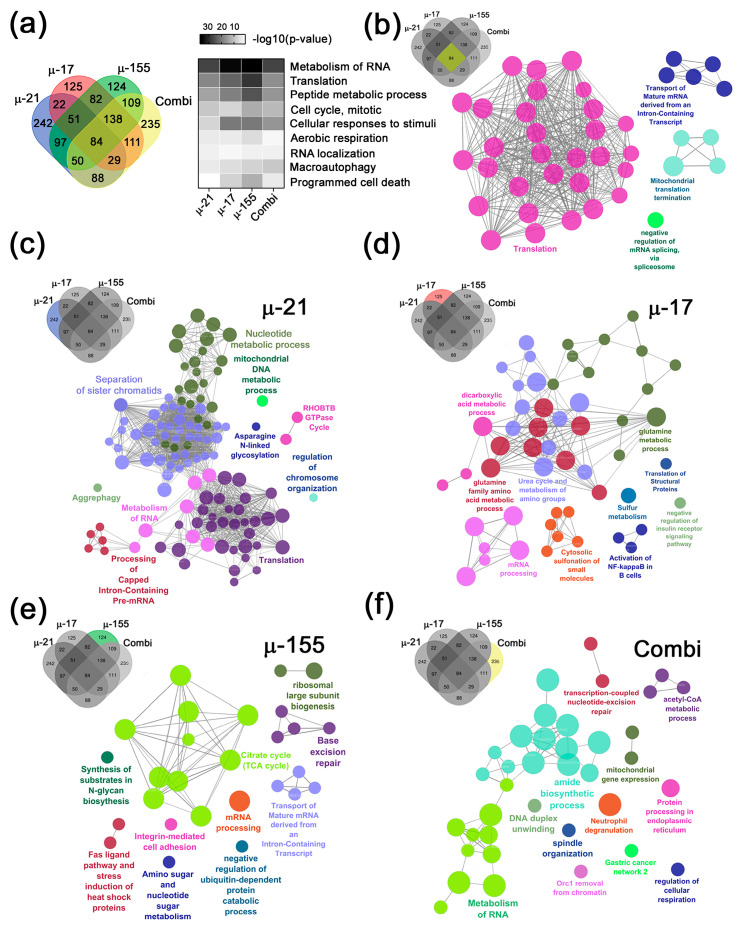
Bioinformatics analysis of proteomic profiling in Caco-2 cells following treatment with µ-ASOs in mono regime or in combination. (**a**) Venn diagram illustrating shared and unique differentially expressed proteins (DEPs) across Caco-2 cells treated with a single µ-ASO (µ-21, µ-17, or µ-155) or combination (Combi: µ-21 + µ-17 + µ-155). DEPs were defined as |log_2_(Fold Change (FC))| ≥ 1, *p*-value ˂ 0.05, and the top 9 common biological processes modulated by all µ-ASOs, either individually or in combination. (**b**) Functional annotation of 84 DEPs modulated by all single µ-ASO and Combi. (**c**–**f**) Functional annotation of biological processes associated with unique DEPs modulated by µ-21, µ-17, µ-155, and Combi, respectively. Functional enrichment analysis was performed using the ClueGO v2.5.9 plugin (Cytoscape), integrating annotations from Gene Ontology (GO), KEGG, and WikiPathways.

At the next stage, we intersected the complete DEP datasets obtained for cells treated with individual miRNA-targeted µ-ASOs (µ-21, µ-17, or µ-155) and their combined treatment (Combi) in order to identify both overlapping and unique proteins for each cohort (Figure 3a). We identified 84 shared proteins differentially expressed in all four miRNA-targeted µ-ASO-treated cohorts, alongside unique targets for each µ-ASO, including 242 specific targets for µ-21, 125 for µ-17, 124 for µ-155, and 235 for Combi (Figure 3a).

First, we performed functional analysis on the 84 shared DEPs and found that these proteins are involved in cytoplasmic and mitochondrial translation as well as mRNA splicing (Figure 3a,b). Subsequently, we turned to the datasets for each individual µ-ASO and conducted functional analyses of the unique proteins identified exclusively in each respective treatment group. Functional annotation of the unique DEP datasets for each µ-ASO revealed a clear separation of protein functional annotations into those performing basic homeostasis functions and those involved in maintaining more cancer-associated terms (Figure 3c–f).

The unique proteins differentially expressed upon µ-21 treatment include those involved in nucleotide and asparagine metabolism, RNA processing, and translation, as well as sister chromatid separation during mitosis and the regulation of chromosome organization (Figure 3c). Transfection with µ-17 results in changes in levels of proteins associated with glutamine, sulfur, and dicarboxylic acid metabolism, translation, mRNA processing, as well as the inflammatory response or the response to oxidative stress (e.g., activation of NF-κB in B-cells) (Figure 3d). Treatment with µ-155 alters the expression of proteins participating in carbohydrate metabolism and the tricarboxylic acid (TCA) cycle, ribosome biogenesis, ubiquitin-dependent protein degradation, mRNA processing and transport, as well as base excision repair, adhesion, and apoptosis via the Fas-ligand pathway (Figure 3e). In turn, Combi-transfection led to changes in DEPs responsible for protein processing, amide metabolism, RNA metabolism, and the regulation of cellular respiration, as well as those participating in DNA replication and repair, and the organization of the mitotic spindle (Figure 3f).

In the subsequent analysis, we did not limit our consideration to unique DEPs identified for each treatment group, as this would provide only a fragmented view of the proteomic response. While unique targets indeed reflect the specific cellular reaction to each individual oligonucleotide and the Combi treatment, they overlook numerous potential protein interactions within the complete sets of DEPs that reconstruct the comprehensive functional landscape of cellular changes for each group. Therefore, all further analyses were performed using the full DEPs datasets, which include 663 DEPs for µ-21, 642 DEPs for µ-17, 735 DEPs for µ-155, and 844 DEPs for Combi. We performed functional annotation of these datasets and intersected the resulting lists of terms to identify common biological functions across all treatment groups. We demonstrated that all µ-ASOs, both upon mono- and combination treatment, modulate DEPs primarily associated with fundamental biological processes, including RNA metabolism and localization, translation, peptide metabolism, cellular response to stimulus, and aerobic respiration (Figure 3a). Notably, among the top-ranked processes, the terms more closely linked to tumorigenic potential were also found, specifically cell cycle regulation, macroautophagy, and programmed cell death (Figure 3a). *p*-value assessment revealed a roughly equivalent high contribution of µ-21, µ-155, and Combi to cell cycle regulation, a greater involvement of µ-155 and Combi in macroautophagy, and a more pronounced role of µ-17 and µ-155 in regulating programmed cell death (Figure 3a and Table S1).

Canonical pathway analysis showed that the identified DEPs participate in key signaling cascades, including MAPK, IL, Hedgehog, NOTCH, WNT, and VEGF (Figure S5). Quantitative assessment demonstrated that MAPK, IL, Hedgehog, and NOTCH pathways were represented by 35–38 proteins each, whereas WNT and VEGF pathways involved 60 and 84 proteins, respectively (Figure S5). These pathways exhibited a near-balanced distribution of up- and downregulated DEPs, with a slight predominance of downregulated proteins (Figure S5). Comparative analysis of pathway-associated DEPs indicated that µ-21 and Combi modulated the fewest number of proteins (approximately half of the total DEPs assigned to each pathway) (Figure S5), with µ-21 and Combi showing similar involvement in WNT, IL, VEGF, and NOTCH signaling, and Combi exhibiting stronger engagement in MAPK and Hedgehog pathways (Figure S5). In contrast, µ-17 and µ-155 displayed the broadest pathway modulation, altering 60–75% of pathway-associated proteins 72 h post-transfection (Figure S5).

The intersection of DEPs linked to top-ranked biological terms and signaling pathways suggested that fundamental cellular processes, such as RNA metabolism, peptide metabolic process, translation, aerobic respiration, and RNA localization, are likely governed by other pathways, as their overlap with DEPs involved in MAPK, IL, Hedgehog, NOTCH, WNT, and VEGF signaling pathways did not exceed 15% (Table S1). Conversely, terms associated with oncogenic potential—cellular response to stimulus, cell cycle, macroautophagy, and programmed cell death—showed 30–50% target overlap, peaking at 71% in select cases (Table S1). Despite its high protein representation, the VEGF pathway had the lowest overlap with functional terms, whereas WNT and Hedgehog signaling emerged as the most prominently involved (Table S1).

#### 2.2.2. Key Functional Protein Modules and Top 50 Altered DEPs in Response to µ-ASOs Targeting miR-21, miR-17, and miR-155

**Figure 4:**
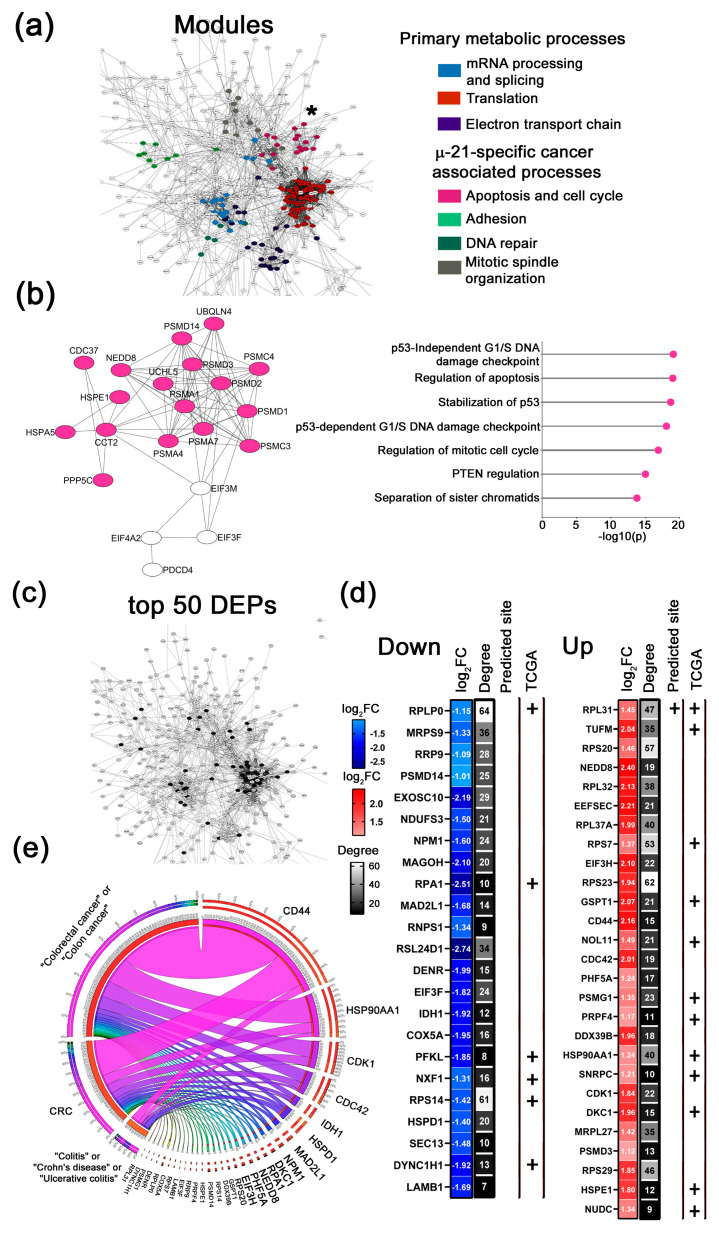
Bioinformatics analysis of the proteomic profiling of Caco-2 cells following µ-21 treatment. (**a**) Protein–protein interaction (PPI) network of differentially expressed proteins (DEPs) identified in Caco-2 cells 72 h post-transfection with µ-21. The network was constructed using the stringApp v2.2.0 plugin in Cytoscape. Edges represent interactions with a STRING score ˃ 0.7 (high confidence). Distinct colors represent functional clusters of DEPs associated with µ-21-specific biological processes in Caco-2 cells (MCODE score ˃ 5). Cluster analysis was performed using the MCODE v2.0.3 plugin in Cytoscape. An asterisk marks the module, which is further detailed in panel B of this figure. (**b**) Module of DEPs involved in apoptosis and cell cycle (left panel). Biological processes regulated by DEPs identified in apoptosis and cell cycle module as determined by ClueGo v2.5.9 plugin (Cytoscape) using annotations from Gene Ontology, KEGG, Wiki Pathways (right panel). (**c**) A fragment of the PPI network demonstrating the topology of the top 50 DEPs evaluated for the µ-21-treated cohort (black nodes). (**d**) top 50 DEPs (down- and upregulated) identified after treatment with µ-21. Plus signs (+) indicate proteins that are either computationally predicted miR-21 targets or correlate with colorectal adenocarcinoma patient overall survival based on TCGA database analysis. Degree represents the number of first neighbors for each protein from the top 50 list in the PPI network. (**e**) Circos plot illustrating text-mining analysis of the top 50 DEPs in µ-21-treated Caco-2 cells, depicting their documented association with colorectal cancer, colitis, ulcerative colitis, and Crohn’s disease. Analysis was conducted using the GenCLiP3 platform.

**Figure 5:**
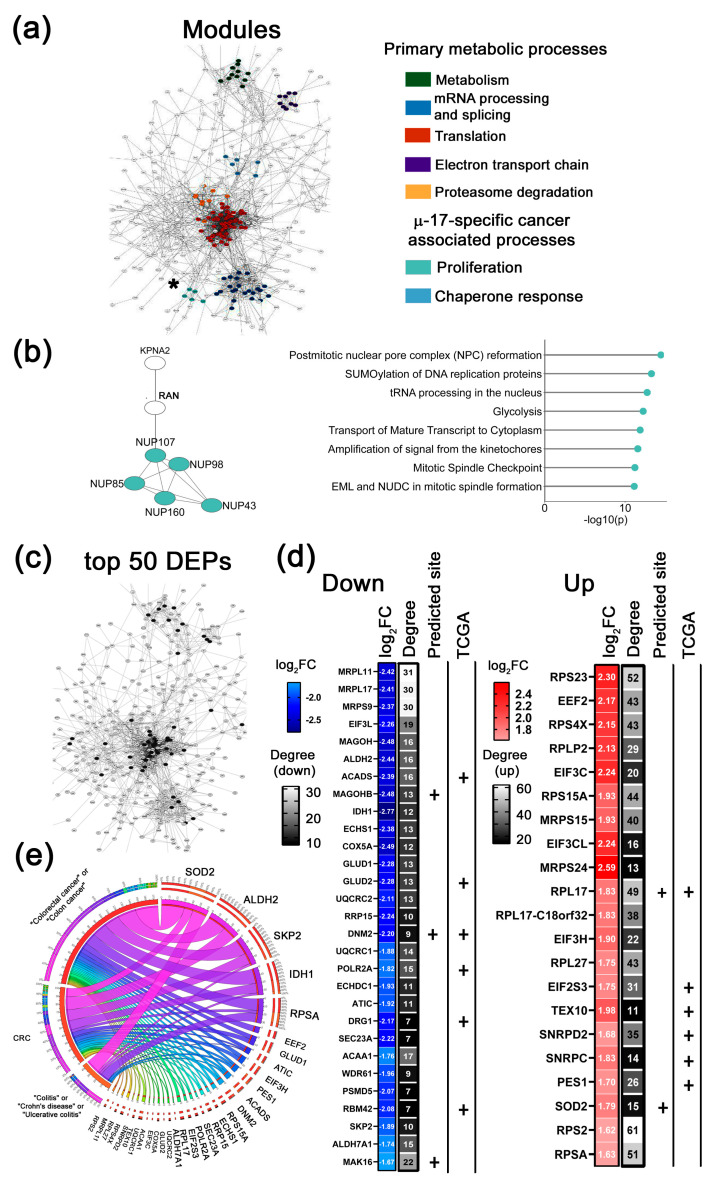
Bioinformatics analysis of the proteomic profiling of Caco-2 cells following µ-17 treatment. (**a**) Protein–protein interaction (PPI) network of differentially expressed proteins (DEPs) identified in Caco-2 cells 72 h post-transfection with µ-17. The network was constructed using the stringApp v2.2.0 plugin in Cytoscape. Edges represent interactions with a STRING score ˃ 0.7 (high confidence). Distinct colors represent functional clusters of DEPs associated with µ-17-specific biological processes in Caco-2 cells (MCODE score ˃ 5). Cluster analysis was performed using the MCODE v2.0.3 plugin in Cytoscape. An asterisk marks the module, which is further detailed in panel b of this figure. (**b**) Module of DEPs involved in proliferation (left panel). Biological processes regulated by DEPs identified in the proliferation module as determined by ClueGo v2.5.9 plugin (Cytoscape) using annotations from Gene Ontology, KEGG, Wiki Pathways (right panel). (**c**) A fragment of the PPI network demonstrating the topology of the top 50 DEPs evaluated for the µ-17-treated cohort (black nodes). (**d**) The top 50 DEPs (down- and upregulated) were identified after treatment with µ-17. Plus signs (+) indicate proteins that are either computationally predicted miR-17 targets or correlate with colorectal adenocarcinoma patient overall survival based on TCGA database analysis. Degree represents the number of first neighbors for each protein from the top 50 list in the PPI network. (**e**) Circos plot illustrating text-mining analysis of the top 50 DEPs in µ-17-treated Caco-2 cells, depicting their documented association with colorectal cancer, colitis, ulcerative colitis, and Crohn’s disease. Analysis was conducted using the GenCLiP3 platform.

**Figure 6:**
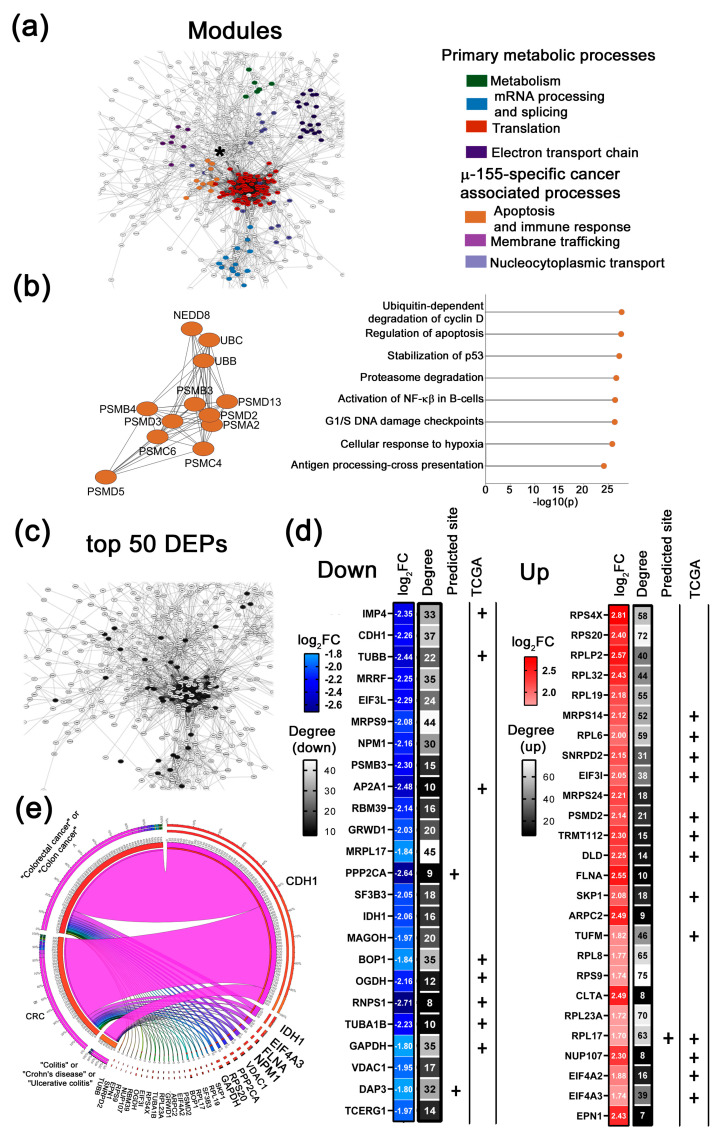
Bioinformatics analysis of the proteomic profiling of Caco-2 cells following µ-155 treatment. (**a**) Protein–protein interaction (PPI) network of differentially expressed proteins (DEPs) identified in Caco-2 cells 72 h post-transfection with µ-155. The network was constructed using the stringApp v2.2.0 plugin in Cytoscape. Edges represent interactions with a STRING score ˃ 0.7 (high confidence). Distinct colors represent functional clusters of DEPs associated with µ-155-specific biological processes in Caco-2 cells (MCODE score ˃ 5). Cluster analysis was performed using the MCODE v2.0.3 plugin in Cytoscape. An asterisk marks the module, which is further detailed in panel b of this figure. (**b**) Module of DEPs involved in apoptosis and immune response (left panel). Biological processes modulated by DEPs from the apoptosis and immune response module, as determined by ClueGo v2.5.9 plugin (Cytoscape) using annotations from Gene Ontology, KEGG, Wiki Pathways (right panel). (**c**) A fragment of the PPI network demonstrating the topology of the top 50 DEPs evaluated for the µ-155-treated cohort (black nodes). (**d**) The top 50 DEPs (down- and upregulated) identified after treatment with µ-155. Plus signs (+) indicate proteins that are either computationally predicted miR-155 targets or correlate with colorectal adenocarcinoma patient overall survival based on TCGA database analysis. Degree represents the number of first neighbors for each protein from the top 50 list in the PPI network. (**e**) Circos plot illustrating text-mining analysis of the top 50 DEPs in µ-155-treated Caco-2 cells, depicting their documented association with colorectal cancer, colitis, ulcerative colitis, and Crohn’s disease. Analysis was conducted using the GenCLiP3 platform.

**Figure 7:**
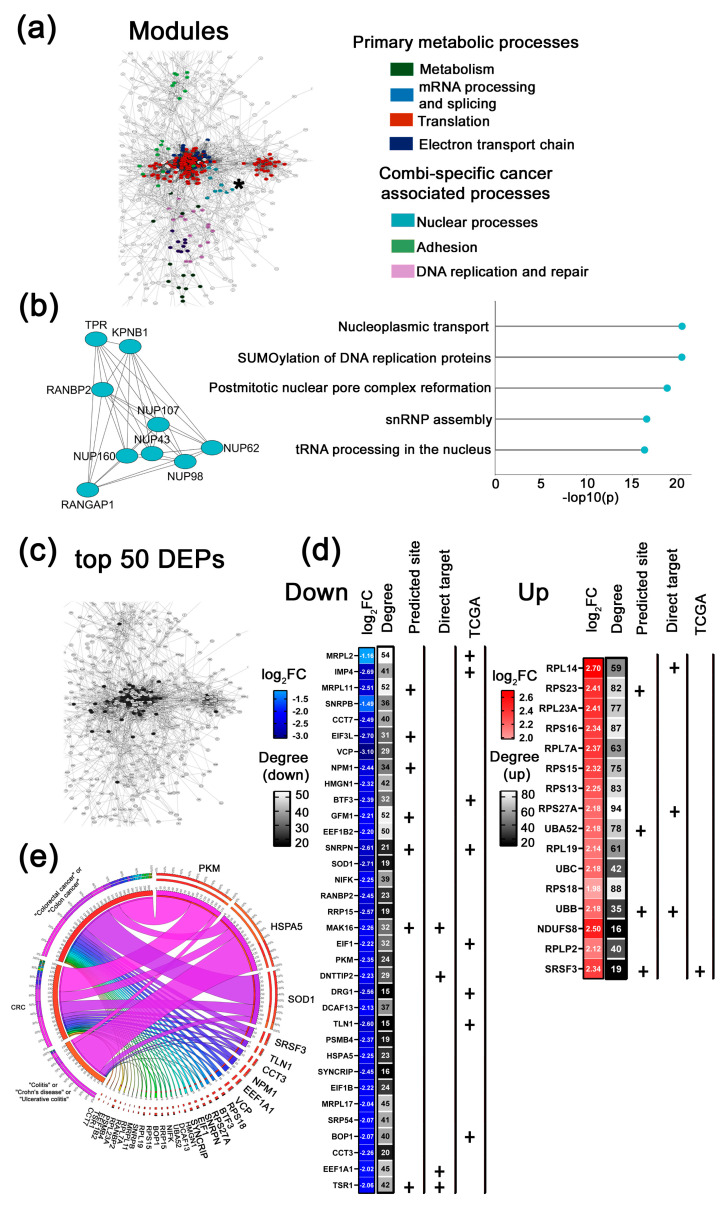
Bioinformatics analysis of the proteomic profiling of Caco-2 cells following Combi treatment. (**a**) Protein–protein interaction (PPI) network of differentially expressed proteins (DEPs) identified in Caco-2 cells 72 h post-transfection with Combi. The network was constructed using the stringApp v2.2.0 plugin in Cytoscape. Edges represent interactions with a STRING score ˃ 0.7 (high confidence). Distinct colors represent functional clusters of DEPs associated with Combi-specific biological processes in Caco-2 cells (MCODE score ˃ 5). Cluster analysis was performed using the MCODE v2.0.3 plugin in Cytoscape. An asterisk marks the module, which is further detailed in panel b of this figure. (**b**) Module of DEPs involved in nuclear processes. Biological processes modulated by DEPs from the nuclear processes module, as determined by ClueGo v2.5.9 plugin (Cytoscape) using annotations from Gene Ontology, KEGG, Wiki Pathways. (**c**) A fragment of the PPI network demonstrating the topology of the top 50 DEPs evaluated for the Combi-treated cohort (black nodes). (**d**) The top 50 DEPs (down- and upregulated) identified after treatment with Combi. Plus signs (+) indicate proteins that are either computationally predicted miR-21/miR-17 or miR-155 targets according to the TargetScan database or directly validated targets of miR-21/miR-17 or miR-155 according to MirTarBase data or correlate with colorectal adenocarcinoma patient overall survival based on the TCGA database. Degree represents the number of first neighbors for each protein from the top 50 list in the PPI network. (**e**) Circos plot illustrating text-mining analysis of the top 50 DEPs in Combi-treated Caco-2 cells, depicting their documented association with colorectal cancer, colitis, ulcerative colitis, and Crohn’s disease. Analysis was conducted using the GenCLiP3 platform.

To delineate the distinct effects of individual µ-ASOs and Combi on the proteomic profile of Caco-2 cells and to identify the most significantly changed proteins associated with the application of miRNA-targeted µ-ASO, we constructed protein–protein interaction (PPI) networks from the full sets of DEPs using the STRING plugin in Cytoscape. We performed functional clustering of DEPs via the MCODE algorithm (score > 5) and revealed densely interconnected protein modules associated with specific biological processes, depicted in color-coded network fragments (Figure 4a, Figure 5a, Figure 6a and Figure 7a). In addition, we ranked all the DEPs in datasets by their degree of protein connectivity together with absolute log_2_ fold-change (|log_2_FC|) values. This systematic approach enabled the identification of the top 50 most differentially expressed and highly interconnected proteins, which are highlighted in black in the respective PPI network panels (Figure 4c, Figure 5c, Figure 6c and Figure 7c).

##### Key Functional Modules Altered by µ-ASOs Treatment

Firstly, we identify groups of highly interconnected proteins in each PPI network and functionally annotate them, revealing the spectrum of biological processes regulated by the constituent proteins. Our comparative approach revealed both similar and unique protein modules in the mono-µ-ASO and Combi-treated groups.

We observed that across all cohorts, based on their functional associations, protein modules segregated into two predominant functional categories: primary metabolic processes and cancer-associated clusters (Figure 4, Figure 5, Figure 6 and Figure 7 and Figures S6–S8). The primary metabolic processes group includes clusters of proteins associated with metabolic reprogramming (Metabolism), the ribosome biogenesis system and general translational activity (Translation), RNA processing (mRNA processing and splicing), and the regulation of proteostasis (Proteasome degradation). These modules are represented by a wide number of molecular targets localized in the central region of the PPI network and exhibit a high degree of interconnectivity (Figure 4a, Figure 5a, Figure 6a and Figure 7a).

The second group of modules includes cancer-associated protein clusters that let us propose more specific effects of each µ-ASO taken alone or in Combi on Caco-2 cells. It should be emphasized that our analysis specifically focused on this group of modules within each cohort examined. Figure 4b, Figure 5b, Figure 6b and Figure 7b depict the modules with the highest MCODE scores among all cancer-associated clusters identified in each cohort, accompanied by a detailed functional annotation of the proteins within the shown module. The structure and functional annotation of the remaining cancer-associated clusters for each group are provided in the Supplementary Materials (Figures S6–S8).

1. µ-21-specific cancer-associated modules

Treatment with µ-21 led to the alteration of DEPs, assembling into modules associated with core survival functions, including apoptosis, cell cycle, adhesion, proliferation, and DNA repair. Among cancer-associated modules, the highest MCODE score was identified for the “apoptosis and cell cycle” cluster (Figure 4b). This module was primarily composed of proteasomal subunits (PSMD1-3; PSMD14; PSMC3-4; PSMA1,5,7), cell cycle regulators (CDC37, NEDD8), the telomere maintenance factor CCT2, as well as chaperones and post-translational modifiers (HSPE1, HSPA5, PPP5C). Functional annotation showed that DEPs in the given module are involved in apoptotic regulation through PTEN-mediated pathways, and cell cycle control via both p53-dependent and independent mechanisms (Figure 4b). Notably, across the DEPs identified for µ-21, we observed the potential upstream regulators of this module, such as the translation initiation factors EIF4A2, EIF3H, and EIF3F, and the tumor suppressor PDCD4 (highlighted as white nodes in Figure 4b). These proteins represent the first neighbors to the components of the “apoptosis and cell cycle” module, and at the same time have been previously directly validated as miR-21 targets according to MirTarBase data.

Moreover, we revealed that the application of µ-21 led to a change in proteins associated with adhesion. The corresponding module consists of laminins (LAMA5, LAMB1, LAMC1) and integrins (ITGA1, ITGAV, ITGA2)—the key proteins regulating extracellular matrix (ECM) attachment and cell motility (Figure S6a). We posit that the anti-migratory effect of µ-21 might be associated with the suppression of key oncoproteins within this cluster, such as the laminin structural components LAMC1 and LAMB1, the cytoskeletal element ACTG1 (γ-actin), and the core matrix glycoprotein FN1 (fibronectin).

Another cluster observed for the µ-21-treated group is the DNA repair module that includes key mismatch recognition proteins (MSH2, MSH6), the single-strand break repair mediator (PARP1), and DNA replication factor (RPA1) (Figure S6a) and highlights the potential impact of µ-21 on genomic stability. Finally, the last observed cluster for µ-21 is mitotic spindle organization module whose composition suggests that µ-21 influences the proper spindle assembly and cell division via regulation of structural components of the centromere–kinetochore complex (CENP-F), cytoskeletal regulators controlling actin/microtubule dynamics (ANLN, TUBB4B) and centrosome maturation factors essential for mitotic exit and cytokinesis (PLK1, CCT5, PFDN4, PFDN6) (Figure S6a). We propose that downregulation of oncogenic proteins within this cluster, including mitotic checkpoint regulator MAD2L1 and the cytoskeletal dynamics regulator TUBB4B, might be vital for the anti-proliferative effect of µ-21.

2. µ-17-specific cancer-associated modules

Analysis of µ-17 PPI network revealed two specific protein clusters responsible for (1) proliferation and (2) chaperone response (Figure 5a,b and Figure S6b). The proliferation-associated module consists of nuclear pore complex (NPC) proteins such as NUP107, NUP98, NUP85, NUP160, and NUP43, which control and maintain mitotic spindle assembly, DNA replication prior to division, as well as post-mitotic reformation of the nuclear pore complex. We suggest that the pronounced alteration of several oncogenic targets within the cluster, including NUP43, NUP160, and the direct miR-17 target NUP98, is likely essential for disrupting subsequent mitotic pore assembly and nucleocytoplasmic transport. Furthermore, cross-referencing with validated miR-17 targets identified two key upstream regulators of this module: KPNA2 and RAN, which function concordantly to mediate nucleocytoplasmic transport through the NPC (Figure 5b, white nodes).

The second µ-17-specific cancer-associated module comprises endoplasmic reticulum (ER) molecular chaperones (PDIA4, PDIA6, GANAB) and heat shock proteins (HYOU1, HSPA5, DNAJB11) (Figure S6b). According to previously published data, µ-17-induced alterations in the profile of these proteins may drive unfolded protein response, autophagy induction, modulation of invasive potential through ER stress-mediated cytoskeletal remodeling, and pro-apoptotic effects [25,26,27] (Figure S6b).

3. µ-155-specific cancer-associated modules

Analysis of the proteomic profile of µ-155-treated cells uncovered a highly significant apoptosis/immune response module, which had the highest MCODE score (Figure 6a,b). While sharing a 15% component overlap with the analogous µ-21 network module (Figure 4b and Figure 6b), the µ-155 cluster was predominantly unique and enriched in core proteasomal components (PSMD3, PSMD5, PSMB4, PSMC4, PSMB3, PSMD13, PSMD2, and PSMA2) and ubiquitin-related proteins (UBC, UBB). Functional characterization demonstrated a strong association with apoptotic regulation, cell cycle control, and immune processes, particularly antigen processing and cross-presentation pathways (Figure 6b).

Two other distinctive clusters exclusive to µ-155 treatment were evaluated as well, including membrane trafficking and nucleocytoplasmic transport (Figure S7). The membrane trafficking module comprises targets mediating clathrin-dependent endocytosis as well as intercellular transport and communication, particularly by means of cell-adhesion molecules (AP2A1, CLTA, and EPN1) (Figure S7).

The nucleocytoplasmic transport-associated cluster comprises two functionally distinct but interconnected domains: core transport machinery represented by nuclear transport receptors (TNPO1, TPR), importins (IPO5, IPO7, IPO9), and regulatory tropomyosins (TPM3, TPM4); and mitotic apparatus components including nuclear pore complex proteins (NUP107, NUP88), microtubule organizers (KIF2C, TPX2), chromosome segregation complex condensin subunits (NCAPG) and central spindle components (RACGAP1) (Figure S7). This integrated clustering highlights a possible coupling between nucleocytoplasmic transport pathways and tumor cell division processes. We suggest that substantial alteration of the most targets within the cluster after application of µ-155, including oncogenic proteins NUP88, RANBP2, NCAPG, IPO5, IPO7, IPO9, and TNPO1, may partially mediate the anti-proliferative effects of µ-155 (Figure S7).

4. Combi-specific cancer-associated modules

Strikingly, DEPs identified upon the Combi treatment were associated with modules that recapitulated yet fundamentally expanded upon those observed in mono treatments. The most significant module, showing the highest MCODE score for Combi, is nuclear processes (Figure 7b). Its core composition integrated components from monotherapy modules—specifically, NPC proteins from µ-17 proliferation cluster (NUP98, NUP107, NUP43, and NUP160) and targets from µ-155 transport module (RANBP2, TPR)—while incorporating unique Combi-specific proteins (NUP62, KPNB1, and RANGAP1). According to the published data, the aforementioned proteins act in a well-coordinated manner within the cell, playing the key roles in controlling mitosis and supporting the idea of a combined regulatory effect of µ-ASOs as part of Combi. In particular, nucleoporin proteins (NUP98, NUP107, NUP43, NUP160, and NUP62) form the nuclear pore complex and are directly involved in its reorganization during mitosis [28]. Among the nucleoporins, the TPR protein stands out: it not only maintains the structural integrity of the pore but also organizes the perinuclear chromatin and influences transcription and gene expression [29]. Meanwhile, transport-related proteins such as KPNB1, RANGAP1, and RANBP2 regulate mitosis through key processes like the import of regulatory factors, spindle assembly, and chromosome organization. Taken together, this integrative module appears capable of influencing nucleocytoplasmic transport by altering the rate of mRNA export from the nucleus, shaping the transcriptional landscape and chromatin architecture, and contributing to mitotic errors and chromosomal instability. Through these mechanisms, it may potentially modulate both the cell cycle and programmed cell death.

Another cancer-associated module, the adhesion cluster (Figure S8), shows conceptual similarity to the µ-21-specific corresponding cluster, though only three integrin family proteins (ITGA1, ITGA2, ITGAV) are shared between them. The Combi adhesion module contains diverse proteins mediating cell–cell contacts and extracellular matrix attachment (TLN1, ITGB1, ITGA6, and SRC), heat shock proteins (DNAJB11, DNAJC9), microtubule components (TUBA1C, TUBA1B), and cytoskeleton-regulating enzymes (PPP2CA), collectively influencing tumor cell migration and adhesion (Figure S8). We suggest that coordinated alteration of a network of pro-invasive oncogenes within this module, such as the integrin ITGA6, the microtubule component TUBA1C, the non-receptor kinase SRC (a master integrator of adhesion, growth, and migration signals), and TLN1 (talin-1), which critically links integrins to the actin cytoskeleton, might in part mediate the anti-migratory effect of treatment.

The DNA replication/repair module, while conceptually similar to that observed for µ-21, shows no protein overlap. In Combi, this cluster contains DNA polymerases (POLD2, POLD3, and POLE3), primase (PRIM1), and mini-chromosome maintenance proteins (MED4, GTF2E2), along with nucleotide repair protein ERCC2 and cell cycle regulator CCNH (Figure S8). We suppose that coordinated alteration of these proteins forms a functional “hub” integrating DNA replication, repair, and transcription, leading to the induction of replicative stress, accumulation of DNA damage, and chromosomal instability. Functionally, this would manifest as a decrease in replication rate, an increase in DNA damage markers, reprogramming of the transcriptional profile, and, most importantly, cell cycle arrest.

To sum up, all µ-ASOs consistently affected core metabolic and translational events, while every single oligonucleotide uniquely targeted distinct cancer-associated processes—apoptosis, cell cycle, proliferation, and DNA repair for µ-21, proliferation and chaperone response for µ-17, and intracellular transport, apoptosis, and immune response regulation for µ-155. The Combi induced a unique effect by integrating these distinct mechanisms into novel, complex networks targeting a broader spectrum of functions simultaneously.

##### Key Altered DEPs upon µ-ASOs Targeting to miR-21, miR-17 and miR-155, Treatment

To identify the key top 50 proteins mediating the effects of individual and combinatorial µ-ASO treatments, we performed an additional analysis of the complete datasets of DEPs for each cohort. All proteins in the full DEP lists were double-ranked according to two parameters: (1) the highest differential expression and (2) the greatest degree of connectivity within the PPI network. The top 50 DEPs were selected for each µ-ASO-treated group.

In Figure 4c, Figure 5c, Figure 6c and Figure 7c, the targets included in the top 50 DEPs for each group are marked in black, allowing for the assessment of their topology within the constructed networks. Notably, most of these targets in all groups are located in the central regions of the PPI networks and co-localize with the primary metabolic modules.

The number and patterns of up- and downregulated proteins observed within each top 50 DEPs list were different for each cohort (Figure 4d, Figure 5d, Figure 6d and Figure 7d). It is important to emphasize that at the analyzed time point, inhibition of oncogenic miRNAs results in proteomic alterations that are largely driven by cascade effects within the protein-protein interaction network, rather than direct derepression of primary targets. This accounts for the observed bidirectional changes in protein levels.

1. top 50 DEPs altered in response to µ-21

We observed that the top 50 DEPs delineated after treatment with µ-21 are represented by 23 down- and 27 upregulated proteins (Figure 4c,d). Among the downregulated targets are several groups of proteins implicated in RNA biogenesis and protein synthesis (RPLP0, MRPS9, NPM1, RPS14, EIF3F, RSL24D1, and DENR), RNA processing and nuclear export (RRP9, NXF1, RNPS1, MAGOH, and EXOSC10), metabolism (IDH1, PFKL, NDUFS3, and HSPD1), cell cycle and DNA replication (RPA1, MAD2L1, and SEC13), cytoskeleton regulation (DYNC1H1, LAMB1) and proteasome degradation (PSMD14) (Figure 4d).

Among the upregulated targets are proteins implicated in translation (RPL31, RPL32, RPL37a, RPS20, RPS7, RPS23, RPS29, TUFM, EEFSEC, EIF3H, GSPT1, DKC1, MRPL27, and NOL11), RNA splicing (PRPF4, DDX39B, PHF5A, and SNRPC), cell cycle and apoptosis (CDK1, CDC42, and NUDC), protein folding (HSP90AA1, PSMD2, HSPE1, and PSMG1) cell adhesion (CD44) and ubiquitin regulation (NEDD8) (Figure 4d). Since most of the top 50 proteins, except RPS14, EIF3F, NDUFS3, SEC13, and NOL11, exhibit mostly oncogenic function in various malignancies, we suggest that µ-21 application leads to dual effects. The observed pattern of upregulated proteins, including mostly ribosomal components and translation initiation factors, leads us to assume that µ-21 may exert compensatory effects. These include enhancing the synthesis and folding of new proteins, as well as facilitating the fine-tuning of proteostasis and helping cells to avoid transitioning into an apoptotic state. At the same time, evaluated downregulation of different oncogenic targets is likely the basis of the observed therapeutic effect of µ-21, which includes inhibition of excessive protein production and RNA processing, normalization of the cell cycle, and a decrease in cancer cell motility.

2. top 50 DEPs altered in response to µ-17

Moving to the µ-17-treated group, we showed that the top 50 DEPs list contains 29 down- and 21 upregulated proteins (Figure 5c,d). Here, the downregulated proteins are connected with metabolic reprogramming (ALDH2, ACADS, ALDH7A1, IDH1, GLUD1, GLUD2, ECHS1, ACAA1, ECHDC1, ATIC, COX5A, UQCRC1, and UQCRC2), ribosome biogenesis (MRPL11, MRPL17, MRPS9, EIF3L, MAGOH, MAGOHB, MAK16, RRP15, and DRG1), transcription and proteostasis (DNM2, POLR2A, SEC23A, WDR61, PSMD5, RBM42, and SKP2) (Figure 5d). Upregulating proteins are mostly connected to translation regulation (RPS23, EEF2, RPS4X RPLP2, EIF3C, RPS15A, MRPS15, EIF3CL, MRPS24, RPL17, RPL17-c18ORF32, EIF3H, RPL27, EIF2S3, TEX10, PES1, RPS2, and RPSA), RNA processing and splicing (SNRPD2, SNRPC) and maintenance of cell homeostasis (SOD2) (Figure 5d). Similarly to the µ-21 group, the majority of the identified proteins exhibit oncogenic functions. Notable exceptions are the tumor suppressor proteins: ALDH2, ACADS, ECHS1, ACAA1, SEC23A, and RBM42. This observed profile suggests that µ-17 enhances the translational activity of cells. Furthermore, it appears to provide a balancing effect on emerging compensatory responses by restraining excessive transcription and subsequent production of new proteins. Additionally, µ-17 significantly contributes to reversing aberrant metabolic reprogramming.

3. top 50 DEPs altered in response to µ-155

In the µ-155-treated cohort, we observed a near-balanced distribution of downregulated (24 DEPs) and upregulated DEPs (26 DEPs) (Figure 6c,d). Similarly to other single oligonucleotides, it was shown that most of downregulated proteins are involved in ribosome biogenesis (IMP4, BOP1, NPM1, EIF3L, MRPS9, MRPL17, GRWD1, PPP2CA, and DAP3), RNA processing and nuclear export (RBM39, SF3B3, RNPS1, TCERG1, and MAGOH), metabolism of lipids and carbohydrates (IDH1, OGDH, and GAPDH), proteostasis and membrane transport (PSMB3, VDAC1) as well as cytoskeleton regulation and adhesion (CDH1, TUBB, TUBA1B, AP2A1, and MRRF) (Figure 6d). Whereas upregulated targets represent components of ribosomes or translation initiation and elongation factors (RPS4X, RPS20, RPLP2, RPL32, RPL19, MRPS14, RPL6, EIF3I, MRPS24, TRMT112, TUFM, RPL8, RPS9, RPL32a, RPL17, EIF4A2, and EIF4A3), proteins involved in nucleocytoplasmic transport and endocytosis (SNRPD2, NUP107, and CLTA), cell motility (FLNA, ARPC2), proteostasis and ubiquitinilation (EPN1, PSMD2, and SKP1) as well as metabolism (DLD) (Figure 6d).

Similarly to µ-21 and µ-17, µ-155 exhibits a dual role: it enhances cellular translational activity by upregulating components of the ribosomal machinery, the ubiquitination system, and intra- and intercellular transport, while simultaneously suppressing excessive protein synthesis, inhibiting cytoskeletal remodeling, reducing migratory potential, and restraining enhanced energy production and nutrient supply for tumor cells.

4. top 50 DEPs altered in response to Combi

In contrast to individually applied oligonucleotides, the Combi treatment demonstrated a pronounced trend towards suppression of protein expression. This is evidenced by the composition of the top 50 DEPs list in this group, which contains 34 downregulated and only 16 upregulated targets (Figure 7c,d).

Downregulated proteins are taking part in rRNA processing or translation initiation (MRPL2, IMP4, MRPL11, EIF3L, NPM1, BTF3, GFM1, EEF1B2, RRP15, MAK16, NIFK, EIF1, DCAF13, SYNCRIP, EIF1B, MRPL17, SRP54, BOP1, EEF1A1, and TSR1), RNA splicing (SNRPN, SNRPB), protein folding (CCT3, CCT7, HSPA5, VCP, and PSMB4), chromatin and cytoskeleton regulation (RANBP2, HMGN1, TLN1, and DNTTIP2), and metabolism (SOD1, DRG1, and PRM).

Upregulated targets are components of ribosomes (RPL14, RPS23, RPL23A, RPS16, RPL7A, RPS15, RPS13, RPS27A, RPL19, RPS18, and RPLP2) ubiquitin proteasome systems (UBA52, UBC, and UBB), RNA splicing system (SRSF3), and metabolism (NDUFS8). Therefore, we reveal that the Combi treatment exerts a dual effect on Caco-2 cells: (1) a compensatory effect, facilitating the production of ribosomal components and enhancing the degradation of hyper-produced proteins, and (2) a beneficial effect, significantly inhibiting translation and RNA splicing processes, modulating chromatin and cytoskeleton organization, and promoting metabolic reprogramming to suppress energy production and induce energy deprivation in tumor cells.

It is important to note that the analysis of the top 50 DEPs list across all oligonucleotides revealed an absence of common, significantly altered targets shared by all four experimental groups, highlighting the distinct mechanisms of action for each compound. Concurrently, several hub proteins were identified, the alteration of which was characteristic of two or three groups treated with µ-ASOs. The observed proteins were primarily represented by ribosomal components or elements of metabolic systems; however, there were also proteins involved in the regulation of translation that might affect cell proliferation, such as NPM1 (common for µ-21, µ-155, and Combi) and BOP1 (common for µ-155 and Combi).

## 3. Discussion

This study investigates the biological response of human colorectal adenocarcinoma Caco-2 cells to the application of µ-ASOs targeting oncogenic miR-21, miR-17, and miR-155, representing the key drivers of CRC pathogenesis. Given the multifunctional roles of miRNAs of interest, we hypothesized that their suppression would cause significant biological effects and alter the expression of a wide range of downstream protein targets. We revealed that both single µ-ASOs and Combi effectively silenced target miRNA (up to 70%), considerably reduced cell proliferation (up to 50% at 120 nM total concentration), and significantly decreased motility (up to 40% in the case of µ-17).

To investigate the molecular mechanisms through which µ-ASOs, targeting miR-21, miR-17, and miR-155, exert their effects, we analyzed the proteomic profile of Caco-2 cells 72 h after transfection. It is important to highlight several key aspects of our findings. (1) The specific time point (72 h post-transfection) was selected to capture protein level responses following peak miRNA suppression, which was observed at 48 h (Figure S2). Consequently, the obtained profile of changes is specific to this time point and may differ significantly at earlier or later stages. (2) At this specific time point (72 h), the observed alterations in protein levels are likely to be a consequence of downstream effects propagating through the protein–protein interaction network, whereas the signal from the direct derepression of primary miRNA targets seems to be less detectable. (3) We propose that the pool of DEPs identified in the Combi group represents the most rapidly responding and sensitive targets to µ-ASO action. This is particularly relevant given that the concentration of each individual miRNA-targeting oligonucleotide is reduced threefold in the triple combination. (4) Finally, according to our data, the primary driver of the observed phenotypic effects of µ-ASO is the suppression of oncogenic targets, rather than the restoration of tumor suppressor protein expression.

Taken together, our findings on the biological activity of each miRNA-targeting oligonucleotide alone and in combination indicate that Caco-2 cells exhibit lower sensitivity to µ-155 compared to µ-21 and µ-17. However, this difference likely reflects not a weaker therapeutic effect per se, but rather a slower response dynamic following µ-155 transfection, which in turn contributes to the overall kinetics of the triple combination. We would like to emphasize that although the impact of the combination on miRNA levels in Caco-2 cells at the analyzed time point may primarily result from µ-17 and µ-21 activity, the proteomic profile of the Combi cohort is clearly µ-155-dependent, representing an integrative yet distinct signature shaped by the concerted action of all three µ-ASOs.

Proteomic changes were assessed relative to the untreated Caco-2 cells, as this baseline avoids potential artifacts associated with empty delivery vehicles or scrambled oligonucleotides. It should be noted that this approach might have some limitations, since it may not fully capture subtle background effects inherent to oligonucleotide delivery and indirect downstream responses. However, using intact cells provides a straightforward reference point for interpreting ASO-specific proteomic alterations.

An important outcome of this study was the absence of a pronounced effect of the mesyl modification itself on the proteomic profile of Caco-2 cells. Comparison of the control oligonucleotide µ-Scr with the miRNA-targeting µ-ASOs (µ-17, µ-155, and µ-21) demonstrated no shared proteins for all four µ-ASO-treated cohorts together with minimal overlap in the pairwise and triple intersection of protein profiles between µ-Scr and each of the miRNA-targeted ASOs. For µ-17 versus µ-Scr, 48 overlapping proteins were identified; none of them belonged to the top 50 DEPs or to the cancer-associated modules defined for µ-17. For µ-155 versus µ-Scr, 55 overlapping proteins were detected, with only two included in the top 50 DEPs of µ-155 (TUFM and FLNA) and none associated with cancer-related modules. Notably, µ-155 induced a significantly stronger reduction in FLNA, compared with µ-Scr. The µ-21 oligonucleotide exhibited the highest degree of overlap with µ-Scr, sharing 117 proteins. Among these, eight were part of the top 50 DEPs, and ten were single representatives of distinct cancer-associated modules regulated by µ-21. However, for six of these eighteen overlapping proteins, the effect of µ-21 significantly differed from that of µ-Scr, supporting the miRNA-specific action of this oligonucleotide. The remaining twelve overlapping proteins were predominantly involved in fundamental cellular processes, including metabolism (PFKL), translation (TUFM, RPS14, and RRP9), proteasome function (PSMA1, PSMA4, and PSMD1), extracellular matrix organization (FN1, LAMA5), and intracellular transport (DYNC1H1, NXF1, and CCT5). These proteins showed weak to moderate connectivity within the protein–protein interaction network, suggesting a limited contribution to the overall functional response.

Further assessment of functional annotations of the evaluated DEPs in miRNA-targeted µ-ASO-treated groups and their established roles in oncogenesis (as oncoproteins or tumor suppressors) suggests that application of µ-ASOs, either individually or in combination, may elicit two distinct types of responses in Caco-2 cells: (1) therapeutically beneficial and (2) compensatory (Table S2).

### 3.1. Therapeutically Beneficial Effects of µ-ASO Targeted to miR-21, miR-17, and miR-155 in Caco-2 Cells

Analysis of the biological functions of the top 50 DEPs from each µ-ASO-treated cohort allows us to evaluate that a potential therapeutically beneficial response may involve metabolic reprogramming that impairs ATP production. We suppose that this energetic reprogramming, associated with µ-ASO application, might occur through modulation of key molecular players involved in (1) glucose and purine metabolism (e.g., PFKL, NDUFS3 (µ-21); ATIC (µ-17); GAPDH (µ-155)) [30,31,32,33,34,35], (2) glutamine synthesis and fatty acid metabolism (e.g., GLUD1, GLUD2, and ACAA1 (µ-17); SF3B3 (µ-155); NIFK, VCP (Combi)) [36,37,38,39,40,41,42], and (3) oxidative phosphorylation (e.g., UQCRC2, UQCRC1 (µ-17); OGDH (µ-155); VCP, GFM1 (Combi)) [43,44]. Notably, µ-21 may influence aberrant serine metabolism by targeting eIF3F [45], while µ-155 could modulate dysregulated asparagine synthesis and arginine consumption through RBM39 [46]. Furthermore, it should be noted that the application of all µ-ASOs appears to be associated with a suppressive effect on the expression of both cytoplasmic and mitochondrial ribosomal components.

Subsequently, we sought to uncover more cancer-specific, therapeutically beneficial effects of µ-ASOs. In particular, it was found that all µ-ASOs were observed to alter proteins involved in key signaling pathways such as NOTCH, Hedgehog, WNT, MAPK, and VEGF. These observations are consistent with previously established data on the involvement of miR-21 in regulating VEGF pathway targets, and miR-155, miR-17 in managing components of WNT signaling in CRC [8,14,18].

In addition, µ-21 may contribute to the regulation of apoptosis via the caspase-3-dependent pathway. In particular, this effect might be related to suppression of several oncogenic proteins, including mitotic spindle assembly checkpoint protein MAD2L1, the exosome complex component EXOSC10, the major replication protein A subunit RPA1, and the translation regulator DENR [47,48,49,50]. Furthermore, µ-21 could potentially inhibit tumor cell proliferation and reduce the immunosuppressiveness of the tumor microenvironment via regulation of microtubule component TUBB4B and the matrix protein LAMB1, respectively [51,52,53].

Application of µ-17 appears to primarily affect proteins associated with cell proliferation such as POLR2A (a core subunit of RNA polymerase II), the mRNA export regulators MAGOH and MAGOHB, and SCF ubiquitin ligase component SKP2 [39,40,41] as long as proteins predicted to be involved in migration, including DNM2 (dynamin-2) and the transcription regulation complex component WDR61, which may influence cytoskeletal remodeling essential for proliferation, invasion and migration [54,55]. Moreover, suppression of miR-17 may be associated with reduced cancer stemness via regulation of the purine synthesis enzyme ATIC and aldehyde dehydrogenase ALDH [52,56,57,58].

µ-ASO-mediated suppression of miR-155, similar to the µ-21 effect, is mostly associated with modulation of proteins controlling programmed cell death. Here, µ-155 may act through alteration of transcription regulators NDAC1 and TCERG1, which could increase mitochondrial membrane permeability [59] and the catalytic subunit of protein phosphatase 2A, PPP2CA, potentially mediating ferroptosis [60]. Moreover, application of µ-155 may suppress pro-inflammatory cytokine production through inhibition of the microtubule component TUBA1B and regulate tumor cell migration through regulation of FLNA (filamin-A) [61].

In turn, application of the Combi is associated with changes in proteins involved in all the aforementioned processes—proliferation, migration, and apoptosis—which appear to involve mechanisms distinct from monotherapy with individual µ-ASOs. We propose that the Combi effect on proliferation may involve inhibition of MYC signaling through management of oncogene NIFK, G2/M cell cycle arrest by downregulation of SNRPN, and disruption of mitotic spindle assembly via inhibition of the chaperonins CCT3 and CCT7 [12,42]. Suppression of migration and induction of apoptosis via caspase-3/7 activation may result from the downregulation of SYNCRIP, a protein implicated in RNA metabolism [62].

All targets mentioned above represent indirect protein targets of miR-21, miR-17, and miR-155. To assess the presence of potential direct targets among the identified DEPs, we performed a two-step analysis. First, we used the TargetScan database to search for predicted binding sites for miR-21, miR-17, and miR-155 within the mRNAs of the top 50 DEPs.

It was shown that these mRNAs within the top 50 DEPs list possess predicted binding sites for the miRNAs of interest and represent their putative targets: RPL31 for µ-21 (Figure 4d); SOD2, MAGOHB, MAK16, RPL17, and DNM2 for µ-17 (Figure 5d); RPL17, PPP2CA, and DAP3 for µ-155 (Figure 6d); as well as SNRPN, UBB, UBA52, SRSF3, MAK16, GFM1, NPM1, EIF3A, and MRPL11 for Combi (Figure 7d).

Second, we employed the miRTarBase database to identify any previously established, directly validated targets of miRNAs of interest (e.g., by PAR-CLIP or dual luciferase assay) among the top 50 DEPs and protein module participants.

It was found that the pool of evaluated DEPs includes direct targets for each miRNA in corresponding cohorts—µ-21: PDCD4, EIF4A2, EIF3M, EIF3F, CCT2, and CDC37; µ-17: NUP98, NUP85, RAN, and KPNA2; µ-155: TNPO1; Combi: MAK16, DNTTIP2, RPL14 and RPS27A, as well as UBB, which all represent direct targets of miR-17.

### 3.2. Compensatory Response of Caco-2 Cells on Treatment with µ-ASO Targeted to miR-21, miR-17 and miR-155

In contrast to the therapeutically beneficial effects, µ-ASOs application was also observed to be associated with compensatory reactions of tumor cells. Both mono and Combi treatments provoked a pronounced upregulation of ribosomal components and specific translation initiation factors (e.g., RPL37a, RPS7, RPS23, and eIF3F (µ-21); RPS2, RPL27, RPS23, and RPS4X (µ-17); RPL17, RPLP2, RPS20, RPL23A, RPL6, RPL8, and RPS9 (µ-155); RPS27A, RPS16, RPS13, RPS15, EIF1, RPS23, and RPL23A (Combi)). We suggest these events may reflect an attempt of tumor cells to counter the energy crisis imposed by metabolic reprogramming and to reconstruct pro-survival pathways to maintain the oncogenic phenotype. Such activation of translation machinery was coupled with an increase in levels of proteasomal subunits PSMD2 (µ-155), PSMD3 (µ-21), components of the ubiquitination machinery UBB, UBA52, UBC (Combi), and a suppression of the proteasome assembly inhibitor PSMD5 (µ-17), indicating a possible concerted effort to maintain proteostatic capacity under stress.

It should be noted that the observed compensatory effects, while unfavorable from a therapeutic perspective, do not represent a negative characteristic of the µ-ASOs themselves. On the contrary, they reveal new opportunities for their application, for example, in combination with agents designed to combat the observed adaptations. For instance, our studies showed that µ-ASOs action can be potentiated by simultaneous treatment with the translation inhibitor cycloheximide (CHX) (Figure S9) [63]. Application of CHX at 0.05 µg/mL, a concentration at which its standalone effect does not exceed 10%, resulted in an additive enhancement of the antiproliferative activity of µ-17, µ-155, and Combi in Caco-2 cells from 45–50% to 75% (Figure S9a,b). Extended dose–response analysis revealed that CHX at 0.025–0.05 µg/mL concentrations demonstrates additive-to-synergistic suppression of Caco-2 cell viability when combined with µ-17 (Bliss and HSA synergy scores of 6.164 and 16.122, respectively), while co-application with µ-155 demonstrated clear synergistic effects (Bliss and HSA synergy scores: 12.364 and 22.235) (Figure S9c,d). These findings indicate that even low-dose CHX can substantially enhance µ-ASO therapeutic efficacy. In addition, co-administration of µ-ASOs with inhibitors of ribosome biogenesis, proteasome assembly antagonists (e.g., bortezomib) [64], ubiquitin-activating enzyme inhibitors (e.g., the Phase I clinical candidate TAK-243 [65] [NCT03816319, NCT06223542]), novel small-molecule inhibitors targeting specific ubiquitin ligase complexes [66], or conventional chemotherapeutic regimens may represent a highly promising strategy.

### 3.3. Correlation with Previously Reported Literature and Clinical Observations

To assess the relevance of the identified top 50 DEPs to CRC pathogenesis, we compared our findings with previously published literature and clinical data. For this purpose, a systematic text-mining analysis was performed for all top 50 DEPs in each group to establish associations between the identified protein targets and well-characterized molecular players involved in CRC, colitis, ulcerative colitis, and Crohn’s disease—highly aggressive pathologies frequently associated with CRC development.

Text-mining analysis for the group treated with µ-21 showed that 30 out of 50 proteins are already described in the context of CRC and associated diseases (Figure 4e). The aforementioned therapeutically beneficial targets regulated by µ-21 include both well-characterized protein effectors in CRC, such as MAD2L1, and proteins with limited prior association with CRC pathogenesis, including eIF3F, NPM1, LAMB1, RPL31, and RPA1. Other mentioned targets, such as PFKL, NDUFS3, DENR, TUBB4B, EXOSC10, RPL37a, RPS7, and RPS23, had no prior literature links to CRC and may be considered as novel diagnostic markers or therapeutic targets of CRC (Table 1).

Similar analysis performed for the group treated with µ-17 revealed that 32 proteins were previously annotated as molecular players in the context of colorectal cancer, colitis, ulcerative colitis, or Crohn’s disease (Figure 5e). Among the key targets evaluated in this study are well-studied proteins in CRC and associated diseases, including ALDH2 and SKP2, moderately to poorly characterized proteins in CRC pathogenesis, such as DNM2, ATIC, POLR2A, GLUD2, RPL17, UQCRC1, and UQCRC2, as well as uncharacterized targets, including WDR61, ACAA1, RPS2, RPL27, RPS23, RPS4X, PSMD5, MAGOH, and MAGOHB (Table 1).

Text-mining analysis of the top 50 DEPs for the group treated with µ-155 revealed that 29 out of 50 proteins have previously documented associations with colorectal cancer, colitis, or Crohn’s disease (Figure 6e). Among them, moderately characterized targets include FLNA, NPM1, GAPDH, and PPP2CA. Poorly studied proteins in the CRC context are represented by BOP1, RPL17, TUBA1B, RBM39, RPL23A, PSMD2, RPS9, and SF3B3 (Figure 6e). The newly established proteins are NDAC1, TCERG1, RPLP2, RPS20, RPL6, and RPL8 (Table 1).

Finally, text-mining analysis of the Combi top 50 DEPs showed that 32 proteins have established associations with colorectal cancer, colitis, or Crohn’s disease (Figure 7e). Among the most promising Combi targets are moderately characterized proteins, such as NPM1, VCP, CCT3, SNRPN, BOP1, SYNCRIP, EIF1, and RPS27A, poorly characterized proteins in the CRC context, including NIFK, UBA52, CCT7, RANBP2, RPL23A, and RPS15, as well as newly identified proteins such as UBB, UBC, RPS16, RPS13, RPL23, and GFM1 (Figure 7e and Table 1).

In addition to the text-mining analysis, for the proteins that contained predicted binding sites for miR-21, miR-17, and miR-155, as well as for those previously validated as direct targets of these miRNAs, we compared the direction of their expression changes (up/down) following µ-ASO treatment with the survival data of patients with colorectal adenocarcinoma (TCGA database). Furthermore, for these proteins, correlations between expression level alterations and colorectal adenocarcinoma tumor stage (I–IV) were assessed using the GEPIA3 platform (Figure S10d,f).

It was found that elevated expression of RPL31, CCT2, RPL17, and NUP85 (Figure S10a,c,e,h), as well as downregulation of NUP98, CDC37, DNM2, and SNRPN resulted from corresponding µ-ASOs treatment, correlating with improved survival in colorectal adenocarcinoma patients according to the TCGA database (Figure S10b,g,i,j). Moreover, the elevated level of CCT2 and RPL17, observed as a result of µ-21 and µ-17 treatment, respectively, might represent a potential mechanism to counteract colon adenocarcinoma progression in later stages, since the decreased expression of these two proteins is associated with advanced colon adenocarcinoma staging, according to GEPIA3 data (Figure S10d,f).

To sum up the analyzed data, we discovered that application of µ-ASOs, both individually and as a Combi, alters the expression of both well-established protein targets in the context of CRC and entirely novel molecular players.

In conclusion, our study provides significant insights for colorectal cancer research and treatment. We confirm existing knowledge about miRNA-regulated pathways in CRC and expand the potential repertoire of therapeutic targets by identifying novel proteins within key functional clusters and the top 50 DEPs lists, which might play a significant role in CRC progression. Moreover, we observed that the combination of three µ-ASOs (Combi) appears to act through a unique mechanism, in contrast to individual oligonucleotides.

Looking forward, our findings highlight a potential therapeutic avenue, considering the application of single µ-ASOs targeting miR-21, miR-17, and miR-155, as well as their Combi as a candidate treatment against CRC. Furthermore, this miRNA-targeted approach could potentially be reinforced by a combination with drugs designed to target adaptive metabolic rewiring and enhanced translation activity, offering a synergistic strategy to overcome treatment resistance and expand the arsenal of effective regimens against CRC.

## 4. Materials and Methods

### 4.1. Oligonucleotide Synthesis

The standard phosphoramidite solid-phase synthesis of all modified and unmodified oligonucleotides was carried out on an ASM-800 DNA/RNA synthesizer (Biosset, Novosibirsk, Russia). Oligonucleotides were synthesized at the 0.4 μmol scale using standard commercial 2-cyanoethyl deoxynucleoside phosphoramidites and CPG solid supports (Glen Research, San Diego, CA, USA). Methylsulfonyl (mesyl, µ) phosphoramidate modifications were introduced via a modified oxidation step, as described previously [22,67]. Oligonucleotides were purified by reversed-phase HPLC using standard gradients of acetonitrile in triethylammonium acetate buffer (pH 7.0), followed by precipitation with LiClO_4_ in acetone and dissolution in deionized water. All oligonucleotides used in the study are listed in Table 2.

### 4.2. Transfection of Tumor Cells with µ-ASOs

Human colorectal adenocarcinoma cells Caco-2 were obtained from the shared research facility “Vertebrate cell culture collection” (Institute of Cytology RAS, St. Petersburg, Russia). Tumor cells were routinely tested for mycoplasma contamination and were confirmed to be mycoplasma-free. Transfection was performed by using Lipofectamine 2000^TM^ (Invitrogen, Carlsbad, CA, USA) according to the manufacturer’s protocol, as described previously [22]. The lipoplexes were pre-formulated in Opti-Mem (Thermo Fisher Scientific, Waltham, MA, USA) for 20 min and added to Caco-2 cells following by incubation at 37 °C in a humidified atmosphere with 5% CO_2_ for 4 h in serum and antibiotic-free Dulbecco’s modified Eagle’s medium (DMEM). The medium was then replaced with DMEM containing 10% fetal bovine serum (FBS) (Thermo Fisher Scientific, Waltham, MA, USA) and 1% antibiotic antimycotic solution (10,000 μg/mL streptomycin, 10,000 IU/mL penicillin, and 25 μg/mL amphotericin) (Biochemist, St. Petersburg, Russia), and the cells were further cultivated for 0–72 h.

### 4.3. Flow Cytometry

Four hours after transfection with FITC-labeled µ-21 oligonucleotide (Table 1), as described above, cells were harvested, washed with saline solution, resuspended, and fixed in 4% formaldehyde in PBS. Cells were analyzed using Novocyte 3000 (ACEA Biosciences, San Diego, CA, USA) flow cytometer (the excitation wavelength was 488 nm, and the emission wavelength was 520 ± 30 nm). The transfection efficiency was estimated as the percentage of cells with green fluorescence exceeding the maximum level of the autofluorescence of untreated cells, and the mean fluorescence intensity of cells was measured in relative fluorescent units (RFU).

### 4.4. Cell Viability Test

The anti-proliferative effect of µ-ASOs in Caco-2 cells was estimated using the MTT test. Cells were seeded in a 96-well plate (7–8 × 10^3^ cells per well) and transfected with single oligonucleotides at a concentration range of 10–150 nM or in triple combination (40 nM of each oligonucleotide), as described above. In the experiment on concurrent treatment of Caco-2 cells with CHX and µ-ASOs, the cells were transfected with µ-ASOs at a concentration range of 10–120 nM, as described above, and 24 h post-transfection, CHX at a concentration range of 0.025–1.75 μg/mL diluted in antibiotic-free DMEM was added to the cells. At 72 h post-transfection with µ-ASOs, MTT solution at a concentration of 5 mg/mL (Sigma-Aldrich, St. Louis, MO, USA) was added to each well at a dilution of 1:10 (vol.) and incubated with the cells for 3 h at 37 °C and 5% CO_2_. Absorbance was measured using the Multiskan RC reader (LabSystems, Atlanta, GA, USA) at 570 nm, and 620 nm was used as the reference wavelength.

The antiproliferative effect of CHX on Caco-2 cells was evaluated by MTT test, as described above, 24 h after incubation with CHX at a concentration range of 0.05–7.5 µg/mL. The type of µ-ASOs and CHX interaction during concurrent treatment was assessed by the Bliss and HSA models using SynergyFinder web application v.3.0 (https://synergyfinder.fimm.fi/ accessed on 28 November 2025). The synergy score <−10 denotes antagonism, −10–10—additive effect, and >10—synergy.

### 4.5. Scratch Assay

Caco-2 cells at a density of 1.2 × 10^6^ per well were seeded in a serum-free DMEM into a 6-well plate. When cell confluency reached 80%, cells were incubated with mytomicin C (Sigma-Aldrich, St. Louis, MO, USA) (15 µg/mL) in antibiotic-free DMEM for 1 h in order to block the proliferation activity of tumor cells. At 1 h post-incubation, the medium was changed to serum-free and antibiotic-free DMEM and transfected with oligonucleotides in mono or combinative regime at a total concentration of 120 nM (40 nM of each ON in triple combination), as described above. At 24 h after transfection, three wound gaps in each well were scratched vertically with a micropipette tip. The floating cells were washed away with sterile phosphate-buffered saline (PBS) twice before adding fresh DMEM. At 0 and 48 h after scratching, the cells were photographed using a phase-contrast microscope (Zeiss Primo Vert, Zeiss, Oberkochen, Germany) and analyzed with ImageJ v. 1.53e (National Institute of Health, Bethesda, MD, USA). Along with the length of each scratch, at least five photographs were taken, and the width of each scratch was calculated as a mean value ± standard error of at least five measurements obtained for one scratch. Further, for each well, the average scratch width for the given time point was calculated as the mean value ± standard error of three independent scratches inflicted in each well. The migration area was estimated as the ratio of the area filled with cells after 48 h to the initial scratch area. The migration rate of the cells was estimated as the degree of wound healing and calculated according to the formula: υ = (1 − Χ) × 100%, where Χ is the ratio of the scratch width at 48 h to the scratch width at 0 h.

### 4.6. Stem-Loop PCR

Total RNA was isolated from Caco-2 cells 48 h post-treatment with oligonucleotides using RIzol Reagent (DIAM, Moscow, Russia) according to the manufacturer’s protocol. cDNA was synthesized using 3 μg of total cellular RNA in 20 μL reaction mixtures containing reverse transcription buffer (1×) (Biolabmix, Novosibirsk, Russia), 50 nM reverse transcription-specific primer (Table 2), and 100 U of M-MuLV-RH reverse transcriptase (Biolabmix, Novosibirsk, Russia). Reverse transcription was performed as previously described [68,69]. The RT and PCR primers used in the study are listed in Table 2. The level of miRNAs was measured using stem-loop qPCR technology [70,71]. PCR amplification was carried out using BioMaster HS-qPCR SYBR Blue mix (Biolabmix, Russia) according to the manufacturer’s protocol. The obtained qPCR data were analyzed by standard Bio-Rad iQ5 v.2.0 software. The ΔΔCt method was used to determine the relative miRNA levels with U6 serving for normalization.

### 4.7. Proteomic Analysis

Caco-2 cells at a density of 10^6^ per well were seeded in a serum-free DMEM into a 6-well plate, and 24 h later were transfected with single oligonucleotides (120 nM) or their triple combination (40 nM of each ON) in complex with Lipofectamine 2000^TM^, as described above. At 72 h post-transfection, cells were lysed in lysis buffer containing 5% SDS in 50 mM TEAB. Detailed protocols for cell lysis, protein digestion, LC separation conditions, and mass spectrometry parameters are provided below. Experiments were performed in three biological and three technical replicates for each group, resulting in nine replicates per condition. Proteomic analysis was performed using the equipment of the “Human Proteome” Core Facility Centre (Institute of Biomedical Chemistry, Moscow, Russia). The mass spectrometry proteomics data have been deposited to the ProteomeXchange Consortium via the PRIDE [72] partner repository with the dataset identifier PXD069826 and 10.6019/PXD069826.

### 4.8. Cell Lysis and Protein Quantification

Transfected Caco-2 cell pellets were lysed in 200 μL of lysis buffer containing 5% SDS in 50 mM TEAB. Samples were sonicated three times for 30 s each on ice using an ultrasonic homogenizer, then centrifuged at 13,000× *g* for 10 min to remove SDS foam. The supernatant was diluted 3-fold with HPLC-grade water for protein quantification using the bicinchoninic acid (BCA) assay (Pierce, Portland, OR, USA) according to the manufacturer’s protocol.

For protein quantification, the prepared BSA standards (0.5–50 μg/μL) or samples (30 μL) were mixed with 1 mL BCA working reagent (containing 1% sodium bicinchoninate, 2% Na_2_CO_3_, 0.16% sodium tartrate, 0.4% NaOH, and 0.95% NaHCO_3_, pH 11.25) and 20 μL 4% CuSO_4_. After mixing, samples were incubated at 56 °C for 20 min in a ThermoMixer Comfort (Eppendorf, Leipzig, Germany), and absorbance was measured at 562 nm using a NanoDrop ND-1000 spectrophotometer (Thermo Fisher Scientific, Waltham, MA, USA) with triplicate measurements.

### 4.9. Sample Preparation for LC–MS/MS Using S-Trap

All buffers and reagents were prepared fresh on the day of analysis, including: S-Trap filters (ProtiFi, Fairport, NY, USA), lysis buffer (10% SDS in 100 mM TEAB), 400 mM chloroacetamide in 50 mM TEAB, 0.5 M TCEP, wash buffer (90% methanol in 100 mM TEAB), 12% phosphoric acid, trypsin solution in 50 mM TEAB, 0.2% formic acid, and 50% acetonitrile/0.1% formic acid. Protein samples (100 μg) were reduced with 2 μL 0.5 M TCEP and alkylated with 4 μL 400 mM chloroacetamide at 80 °C for 30 min, followed by precipitation using 12% phosphoric acid (10% *v*/*v*) and 6× volume wash buffer. After loading onto S-Trap filters (170 μL aliquots, 4000× *g*, 3 min) and three washes with 150 μL wash buffer, on-filter digestion was performed with 40 μL trypsin (1:50 enzyme–protein ratio) at 47 °C for 2 hr. Peptides were eluted sequentially with 80 μL each of 50 mM TEAB, 0.2% formic acid, and 50% acetonitrile/0.1% formic acid, with pooled eluates dried in a vacuum concentrator at 45 °C.

### 4.10. LC–MS/MS Analysis

Peptides were analyzed using an Ultimate 3000 RSLCnano system coupled to a Q-Exactive HF-X mass spectrometer (Thermo Fisher Scientific, Waltham, MA, USA ). Samples (1 μL) were loaded onto an Acclaim μ-Precolumn (0.5 × 3 mm, 5 μm) at 10 μL/min for 4 min, then separated on a Peaky Efficiency FE column (100 μm × 30 cm, 1.9 μm; Molecta) using the following—Mobile phase A: 0.1% formic acid; Mobile phase B: 80% acetonitrile/0.1% formic acid; Gradient: 2–35% B (68 min), 35–99% B (2 min), 99% B (2 min), re-equilibration to 2% B (3 min). Flow rate: 0.3 μL/min. MS parameters were as follows—Ionization: Positive mode NSI; Capillary temperature: 240 °C; Spray voltage: 2.1 kV; MS1: 300–1500 *m*/*z*, 120,000 resolution; MS2: top 40 precursors (z = 2–6+), 15,000 resolution; NCE = 29; Isolation window: ±1 Da; Dynamic exclusion: 90 sec; AGC targets: 1e6 (MS1), 2e5 (MS2).

### 4.11. Data Processing and Analysis

Protein identification was performed using MaxQuant software (v2.0.3.0) with the Andromeda search engine against the UniProt human proteome database (UP000005640, May 2023 release). The search parameters included trypsin digestion with a maximum of two missed cleavages, mass tolerances of ±4.5 ppm for precursor ions and ±20 ppm for fragment ions, and consideration of the following post-translational modifications: carbamidomethylation of cysteine (fixed), oxidation of methionine (variable), and *N*-terminal acetylation (variable). The “match between runs” feature was enabled to enhance identification across samples. All identifications were filtered at a 1% false discovery rate (FDR) at the peptide-spectrum match, peptide, and protein levels, with a minimum requirement of two unique peptides per protein for confident identification.

For quantitative analysis, label-free quantification (LFQ) intensities were log2-transformed and median-normalized. Potential contaminants and false-positive identifications were removed, and only proteins detected with at least two peptides in ≥85% of replicates within any experimental group were retained. Missing values were imputed based on a normal distribution (width = 0.3, downshift = 1.8). Unsupervised pattern recognition was performed via hierarchical clustering and principal component analysis (PCA) using default parameters in Perseus (v2.0.11). Outlier detection based on PCA and hierarchical clustering identified one sample each from the Control, µ-155, and Combi groups that clustered with non-corresponding experimental groups; these samples were excluded from downstream quantitative analysis. Differential expression analysis was conducted using Student’s *t*-test with Benjamini–Hochberg correction, and proteins with |log_2_(FC)| ≥ 1 and adjusted *p*-value ˂ 0.05 were considered significant.

### 4.12. Functional Analysis and PPI Network Reconstruction

Differentially expressed protein (DEP) datasets were obtained by comparing protein expression profiles across µ-ASO-treated groups (µ-Scr, µ-21, µ-17, µ-155, or Combi) and the Control cohort representing the proteome of intact Caco-2 cells with selection criteria of |log_2_FC| ≥1 and adj. *p*-value <0.05. Volcano plots were constructed using Perseus v.2.0.11 software. Venn diagram analysis was performed using the DrawVennDiagram tool (https://bioinformatics.psb.ugent.be/webtools/Venn/ accessed on 16 June 2025).

Functional annotation of the revealed DEPs and reconstruction of PPI networks based on the DEPs datasets obtained for each µ-ASO-treated group were performed using the ClueGo v2.5.9 plugin [73] and Search Tool for the Retrieval of Interacting Genes/Genomes (STRING) database, respectively, in Cytoscape v.3.10.3, according to the previously described procedures [74].

To assess pathway involvement in the top 9 cellular functions identified through functional analysis across all miRNA-targeting µ-ASO-transfected groups, we calculated the ratio of proteins shared between each signaling pathway and cellular function to the total number of proteins within this pathway (%).

The module analysis was performed using the MCODE v2.0.3 plugin. The modules with the MCODE score ≥ 5 were included in the study. The modules were separated into two distinct groups: primary metabolic processes and µ-ASO-specific cancer-associated processes.

### 4.13. Evaluation of Top 50 DEPs and Text Mining Analysis

The top 50 DEPs for each µ-ASO group were selected based on their expression (|log_2_(FC)|) and topology (degree in PPI networks) characteristics. To analyze the co-occurrence of proteins of interest and keywords associated with colorectal cancer and corresponding pathologies in scientific texts deposited in the MEDLINE database, a data-mining analysis of scientific literature was performed using the GenClip3 web service (accessed on 23 May 2025). [75]. The lists of identified top 50 DEPs for each group were uploaded into GenClip3, and a search of the co-occurrence of identified DEPs with the following keywords was performed: colorectal cancer, colon cancer, CRC, ulcerative colitis, colitis, and Crohn’s disease. The results were visualized using Circos [76].

For the top 50 DEPs, the miRNA target site analysis was performed. Experimentally validated targets of miRs of interest were retrieved from miRTarBase v.2025 [77], based on deposited data of high-throughput sequencing of crosslinking immunoprecipitation (HITS-CLIP), photoactivatable-ribonucleoside-enhanced crosslinking (PAR-CLIP), or luciferase reporter assays. Computational predictions were performed using TargetScanHuman v.8.0 web tool, analyzing conserved 8mer, 7mer, and 6mer seed matches [78].

### 4.14. Survival Analysis and Tumor Grade Correlation of DEPs

To analyze the involvement of identified DEPs in the progression of colorectal adenocarcinoma (COAD), analysis of survival rates and their correlations with the expression of studied proteins was performed, and Kaplan–Meier survival curves depending on mRNA expression level were constructed based on The Cancer Genome Atlas (TCGA) clinical data. The violin plots showing expression of studied proteins in tumor cells of patients with COAD grade 1–4 were constructed using Gene Expression Profiling Interactive Analysis 2 (GEPIA2) web service (accessed on 18 April 2025) [79].

### 4.15. Statistics

The data related to the biological performance of µ-ASOs in Caco-2 cells (PCR, MTT-test, and scratch assay results) were statistically processed using one-way ANOVA with post-hoc Tukey test; *p* < 0.05 was considered to be significant.

## Figures and Tables

**Table 1:** Association of top 50 DEPs altered upon treatment with µ-ASO targeted to miR-21, miR-17, and miR-155 with CRC and associated diseases according to text-mining analysis.

**µ-21**		
**Well-Studied**	**Moderate-to-Low Association with CRC and Associated Diseases**	**No Prior Links** **(Emerging Role in CRC)**
CD44; HSP90AA1; CDK1; IDH1; HSPD1; MAD2L1	eIF3F; NPM1; LAMB1; RPL31; RPA1; DKC1; NEDD8; DYNC1H1; RPLP0; RRP9; PSMD14; RPS20; EIF3H; GSPT1; PHF5A; PSMG1; PRPF4; DDX39B; DENR; COX5A; RPS14; HSPE1	PFKL; NDUFS3; DENR; TUBB4B; EXOSC10; RPL37a; RPS7; RPS23; NUDC; SEC13; MRPS9; RPL32; EEFSEC; MAGOH; NOL11; RNPS1; RSL24D1; SNRPC; NXF1; MRPL27; PSMD3; RPS29; TUFM
**µ-17**		
**Well-Studied**	**Moderate-to-Low Association with CRC and Associated Diseases**	**No Prior Links** **(Emerging Role in CRC)**
RPSA; IDH1; SKP2; ALDH2; SOD2	RPS2; MRPL11; RPL27; RPS4X; SNRPD2; TEX10; UQCRC1; ACAA1; EIF3C; COX5A; GLUD2; UQCRC2; ALDH7A1; RPL17; EIF2S3; POLR2A; SEC23A; RRP15; ECHS1; RPS15A; DNM2; ACADS; PES1; EIF3H; ATIC; GLUD1; EEF2	RPS23; MRPL17; MRPS9; RPLP2; IF3L; MAGOH; MRPS15; IF3CL; MRPS24; MAGOHB; RPL17-C18orf32; ECHDC1; SNRPC; DRG1; WDR61; PSMD5; RBM42; MAK16
**µ-155**		
**Well-Studied**	**Moderate-to-Low Association with CRC and Associated Diseases**	**No Prior Links** **(Emerging Role in CRC)**
CDH1; VDAC1; NPM1; FLNA; EIF4A3; IDH1	TUBB; SNRPD2; EPN1; RPS9; NUP107; RBM39; OGDH; EIF3I; RPS4X; TUBA1B; RPL23A; GRWD1; ARPC2; EIF4A2; PSMD2; BOP1; RPL17; SKP1; SF3B3; RPL19; GAPDH; RPS20; PPP2CA	RPLP2; RPL32; IMP4; MRPS14; MRRF; EIF3L; MRPS9; RPL6; MRPS24; PSMB3; TRMT112; DLD; AP2A1; MRPL17; TUFM; RPL8; MAGOH; RNPS1;CLTA; DAP3; TCERG1
**Combi**		
**Well-Studied**	**Moderate-to-Low Association with CRC and Associated Diseases**	**No Prior Links** **(Emerging Role in CRC)**
RPS18; VCP; EEF1A1; NPM1; CCT3; TLN1; SRSF3; SOD1; HSPA5; PKM	CCT7; TSR1; EEF1B2; PSMB4; RPL23A; RANBP2; RPL7A; MRPL11; SNRPB; RPL19; RPS15; BOP1; RRP15; NIFK; UBA52; DCAF13; HMGN1; SYNCRIP; EIF1; SNRPN; RPS27A; BTF3	RPL14; MRPL2; RPS23; IMP4; RPS16; EIF3L; RPS13; GFM1; MAK16; UBC; DNTTIP2; UBB; DRG1; NDUFS8; RPLP2; EIF1B; MRPL17; SRP54

**Table 2:** Oligonucleotides and primers used in the study.

**ASO**	**Sequence 5′-3′**
μ-17	CμTμAμCμCμTμGμCμAμCμTμGμTμAμAμGμCμAμCμTμTμTμG
μ-21	TμCμAμAμCμAμTμCμAμGμTμCμTμGμAμTμAμAμGμCμTμA
μ-155	AμAμCμCμCμCμTμAμTμCμAμCμGμAμTμTμAμGμCμAμTμTμAμA
μ-Scr	CμAμAμGμTμCμTμCμGμTμAμTμGμTμAμGμTμGμGμTμT
FITC-μ-21	FITC-TμCμAμAμCμAμTμCμAμGμTμCμTμGμAμTμAμAμGμCμTμA
**Primer**	**Sequence 5′-3′**
**Reverse transcription primers**	
RT-mir-17	GTCGTATCCAGTGCAGGGTCCGAGGTATTCGCACTGGATACGACCTACCTGCAC
RT-mir-155	GTCGTATCCAGTGCAGGGTCCGAGGTATTCGCACTGGATACGACGACACCCCTATCA
RT-mir-21	GTCGTATCCAGTGCAGGGTCCGAGGTATTCGCACTGGATACGACTCAACATCAG
RT-U6	GTCGTATCCAGTGCAGGGTCCGAGGTATTCGCACTGGATACGACAAAAATATGGAACG
**PCR primers**	
mir-17-F	AGACAAAGTGCTTACAGTGC
mir-155-F	ACTTAATGCTAATTGTGATAGG
mir-21-F	AGACTAGCTTATCAGACTGA
U6-F	CTCGCTTCGGCAGCACA
Universal reverse	GTGCAGGGTCCGAGGT

μ—methanesulfonyl phosphoramidate modification of internucleotidic bond.

## Data Availability

The original data presented in the study are openly available in the PRIDE repository with the dataset identifier PXD069826 and DOI: 10.6019/PXD069826.

## Supplementary Materials

The supporting information can be downloaded at: https://www.mdpi.com/article/10.3390/ijms262311747/s1.

## Abbreviations

The following abbreviations are used in this manuscript:

µ-ASOs	Methanesulfonyl phosphoramidate-modified antisense oligonucleotides
ACAA1	Acetyl-CoA Acyltransferase 1
ACADS	Acyl-CoA Dehydrogenase, Short Chain
ACTG	Gamma-actin
ALDH2	Aldehyde Dehydrogenase 2
ALDH7A1	Aldehyde Dehydrogenase 7 Family Member A1
ANLN	Anillin, Actin Binding Protein
AP2A1	Adaptor Related Protein Complex 2 Subunit Alpha 1
ARPC2	Actin-Related Protein 2/3 Complex Subunit 2
ATIC	5-Aminoimidazole-4-Carboxamide Ribonucleotide Formyltransferase/IMP Cyclohydrolase
BCA	Bicinchoninic acid
BOP1	Block of Proliferation 1 Ribosome Biogenesis Factor
BTF3	Basic Transcription Factor 3
CCT2	Chaperonin Containing TCP1 Subunit 2
CCT3	Chaperonin Containing TCP1 Subunit 3
CCT5	Chaperonin Containing TCP1 Subunit 5
CCT7	Chaperonin Containing TCP1 Subunit 7
CAF	Cancer-Associated Fibroblasts
CADM2	Cell Adhesion Molecule 2
CCNH	Cyclin H
CDC37	Cell Division Cycle 37
CDC42	Cell Division Cycle 42
CD44	CD44 Molecule (Cell Surface Glycoprotein)
CDH1	Cadherin 1 (E-Cadherin)
CDK1	Cyclin-dependent kinase 1
CENPF	Centromere protein F
CHX	Cycloheximide
CLTA	Clathrin Light Chain A
COAD	Colorectal adenocarcinoma
COX5A	Cytochrome c Oxidase Subunit 5A
CRC	Colorectal Cancer
DAP3	Death-Associated Protein 3
DCAF13	DDB1 And CUL4 Associated Factor 13
DDX39B	DEAD-Box Helicase 39B
DEPs	Differentially Expressed Proteins
DENR	Density Regulated Re-initiation and Release Factor
DLD	Dihydrolipoamide Dehydrogenase
DKC1	Dyskerin Pseudouridine Synthase 1
DNA	Deoxyribonucleic acid
DNM2	Dynamin-2
DNAJB11	DnaJ Heat Shock Protein Family (Hsp40) Member B11
DNAJC9	DnaJ Heat Shock Protein Family (Hsp40) Member C9
DRG1	Developmentally Regulated GTP Binding Protein 1
DYNC1H1	Dynein Cytoplasmic 1 Heavy Chain 1
ECHS1	Enoyl Coenzyme A Hydratase, Short Chain, 1
EEF1A1	Eukaryotic Translation Elongation Factor 1 Alpha 1
EEF1B2	Eukaryotic Translation Elongation Factor 1 Beta 2
EEF2	Eukaryotic Translation Elongation Factor 2
EEFSEC	Eukaryotic Elongation Factor, Selenocysteine-specific
ECM	Extracellular Matrix
EIF1	Eukaryotic Translation Initiation Factor 1
EIF1B	Eukaryotic Translation Initiation Factor 1B
EIF2S3	Eukaryotic Translation Initiation Factor 2 Subunit 3
EIF3A	Eukaryotic Translation Initiation Factor 3 Subunit A
EIF3C	Eukaryotic Translation Initiation Factor 3 Subunit C
EIF3CL	Eukaryotic Translation Initiation Factor 3 Subunit C-Like
EIF3F	Eukaryotic Translation Initiation Factor 3 Subunit F
EIF3H	Eukaryotic Translation Initiation Factor 3 Subunit H
EIF3L	Eukaryotic Translation Initiation Factor 3 Subunit L
EIF3M	Eukaryotic Translation Initiation Factor 3 Subunit M
EIF4A2	Eukaryotic Translation Initiation Factor 4A2
EIF4A3	Eukaryotic Translation Initiation Factor 4A3
EPN1	Epsin 1
ER	Endoplasmic Reticulum
ERCC2	Excision Repair Cross-Complementing 2
EXOSC10	Exosome Component 10
FC	Fold change
FLNA	Filamin A
FN1	Fibronectin 1
FOXO3a	Forkhead Box O3a
GANAB	Glucosidase II Alpha Subunit
GAPDH	Glyceraldehyde-3-Phosphate Dehydrogenase
GFM1	G Elongation Factor, Mitochondrial 1
GLUD1	Glutamate Dehydrogenase 1
GLUD2	Glutamate Dehydrogenase 2
GSPT1	G1 to S Phase Transition 1/eRF3a
GRWD1	Glutamate Rich WD Repeat Containing 1
GTF2E2	General Transcription Factor IIE Subunit 2
HMGN1	High Mobility Group Nucleosomal Binding Domain 1
HPLC	High performance liquid chromatography
HSBP2	Heat Shock Protein Family B (Small) Member 2
HSPD1	Heat Shock Protein Family D (Hsp60) Member 1
HSPA5	Heat Shock Protein Family A (Hsp70) Member 5 (BiP/GRP78)
HSPE1	Heat Shock Protein Family E (Hsp10) Member 1
HSP90AA1	Heat Shock Protein 90 Alpha Family Class A Member 1
HYOU1	Hypoxia Upregulated 1
IDH1	Isocitrate Dehydrogenase 1 (NADP+)
IL	Interleukin
IMP4	IMP U3 Small Nucleolar Ribonucleoprotein 4
IPO5	Importin 5
IPO7	Importin 7
IPO9	Importin 9
ITGA1	Integrin Alpha 1
ITGA2	Integrin Alpha 2
ITGA6	Integrin Alpha 6
ITGAV	Integrin Alpha V
ITGB1	Integrin Subunit Beta 1
JAK2	Janus Kinase 2
JNK	c-Jun N-terminal Kinase
KPNA2	Karyopherin Subunit Alpha 2
KPNB1	Karyopherin Subunit Beta 1
LAMA5	Laminin Subunit Alpha 5
LAMB1	Laminin Subunit Beta 1
LAMC1	Laminin Subunit Gamma 1
MAD2L1	Mitotic Arrest Deficient 2 Like 1
MAGOH	Mago Homolog
MAGOHB	Mago Homolog B
MAK16	MAK16 Ribosome Biogenesis Factor
MAPK	Mitogen-Activated Protein Kinase
MED4	Mediator Complex Subunit 4
miRNA, miR	Micro ribonucleic acid
MRPL2	Mitochondrial Ribosomal Protein L2
MRPL11	Mitochondrial Ribosomal Protein L11
MRPL17	Mitochondrial Ribosomal Protein L17
MRPL27	Mitochondrial Ribosomal Protein L27
MRPS9	Mitochondrial Ribosomal Protein S9
MRPS14	Mitochondrial Ribosomal Protein S14
MRPS15	Mitochondrial Ribosomal Protein S15
MRPS24	Mitochondrial Ribosomal Protein S24
MRRF	Mitochondrial Ribosome Recycling Factor
MSH2	MutS Homolog 2
MSH6	MutS Homolog 6
NDUFS3	NADH: Ubiquinone Oxidoreductase Core Subunit S3
NDUFS8	NADH: Ubiquinone Oxidoreductase Core Subunit S8
NEDD8	Neural precursor cell Expressed Developmentally Down-regulated 8
NIFK	Nucleolar Protein Interacting with the FHA Domain and Ki67
NOL11	Nucleolar Protein 11
NPM1	Nucleophosmin 1
NPC	Nuclear Pore Complex
NUDC	Nuclear Distribution C, Dynein Complex Regulator
NUP43	Nucleoporin 43
NUP85	Nucleoporin 85
NUP98	Nucleoporin 98
NUP107	Nucleoporin 107
NUP160	Nucleoporin 160
NXF1	Nuclear RNA Export Factor 1
OGDH	Oxoglutarate Dehydrogenase
PARP1	Poly (ADP-Ribose) Polymerase 1
PBS	Phosphate buffered saline
PCA	Principal component analysis
PCR	Polymerase chain reaction
PFDN4	Prefoldin Subunit 4
PFDN6	Prefoldin Subunit 6
PDCD4	Programmed Cell Death Protein 4
PDIA4	Protein Disulfide Isomerase Family A Member 4
PDIA6	Protein Disulfide Isomerase Family A Member 6
PES1	Pescadillo Ribosomal Biogenesis Factor 1
PFKL	Phosphofructokinase, Liver Type
PHF5A	PHD Finger Protein 5A
PLK1	Polo Like Kinase 1
POLD2	DNA Polymerase Delta 2, Accessory Subunit
POLD3	DNA Polymerase Delta 3, Accessory Subunit
POLE3	DNA Polymerase Epsilon 3, Accessory Subunit
POLR2A	RNA Polymerase II Subunit A
PPI	Protein-protein interaction
PPP2CA	Protein Phosphatase 2 Catalytic Subunit Alpha
PPP5C	Protein Phosphatase 5 Catalytic Subunit
PRIM1	DNA Primase, Catalytic Subunit 1
PRPF4	Pre-MRNA Splicing Tri-SnRNP Complex Factor 4
PTEN	Phosphatase And Tensin Homolog
PSMB3	Proteasome Subunit Beta 3
PSMB4	Proteasome Subunit Beta 4
PSMG1	Proteasome Assembly Chaperone 1
PSMD1	Proteasome 26S Subunit, Non-ATPase 1
PSMD2	Proteasome 26S Subunit, Non-ATPase 2
PSMD3	Proteasome 26S Subunit, Non-ATPase 3
PSMD5	Proteasome 26S Subunit, Non-ATPase 5
PSMD14	Proteasome 26S Subunit, Non-ATPase 14
PSMC	Proteasome 26S Subunit, ATPase
PSMA	Proteasome 20S Subunit Alpha
RACGAP1	Rac GTPase Activating Protein 1
RAN	Ras-Related Nuclear Protein
RANBP2	RAN Binding Protein 2
RANGAP1	Ran GTPase Activating Protein 1
RAS	Rat sarcoma virus
RASA1	RAS p21 Protein Activator 1
RBM39	RNA Binding Motif Protein 39
RBM42	RNA Binding Motif Protein 42
RFU	Relative fluorescence unit
RHOA	Ras Homolog Family Member A
RNA	Ribonucleic acid
RNPS1	RNA Binding Protein With Serine Rich Domain 1
RPA	Replication protein A
RPL14	Ribosomal Protein L14
RPL17	Ribosomal Protein L17
RPL19	Ribosomal Protein L19
RPL23A	Ribosomal Protein L23a
RPL27	Ribosomal Protein L27
RPL31	Ribosomal Protein L31
RPL32	Ribosomal Protein L32
RPL37a	Ribosomal Protein L37a
RPL6	Ribosomal Protein L6
RPL7A	Ribosomal Protein L7a
RPL8	Ribosomal Protein L8
RPLP0	Ribosomal Protein Lateral Stalk Subunit P0
RPLP2	Ribosomal Protein Lateral Stalk Subunit P2
RPS2	Ribosomal Protein S2
RPS4X	Ribosomal Protein S4, X-Linked
RPS7	Ribosomal Protein S7
RPS9	Ribosomal Protein S9
RPS13	Ribosomal Protein S13
RPS14	Ribosomal Protein S14
RPS15	Ribosomal Protein S15
RPS15A	Ribosomal Protein S15A
RPS16	Ribosomal Protein S16
RPS18	Ribosomal Protein S18
RPS20	Ribosomal Protein S20
RPS23	Ribosomal Protein S23
RPS27A	Ribosomal Protein S27a
RPS29	Ribosomal Protein S29
RPSA	Ribosomal Protein SA
RRP9	Ribosomal RNA Processing 9
RRP15	Ribosomal RNA Processing 15
RSL24D1	Ribosomal L24 Domain Containing 1
RT	Reverse Transcription
RUNX3	RUNX Family Transcription Factor 3
SDS	Sodium Lauryl Sulfate
SEC13	SEC13 Homolog, Nuclear Pore and COPII Component
SEC23A	SEC23 Homolog A, COPII Coat Complex Component
SF3B3	Splicing Factor 3b Subunit 3
SKP1	S-Phase Kinase Associated Protein 1
SKP2	S-Phase Kinase Associated Protein 2
SNRPB	Small Nuclear Ribonucleoprotein Polypeptide B
SNRPD2	Small Nuclear Ribonucleoprotein D2
SNRPC	Small Nuclear Ribonucleoprotein Polypeptide C
SNRPN	Small Nuclear Ribonucleoprotein Polypeptide N
SOD1	Superoxide Dismutase 1
SOD2	Superoxide Dismutase 2
SRC	SRC Proto-Oncogene, Non-Receptor Tyrosine Kinase
SRP54	Signal Recognition Particle 54
SRSF3	Serine And Arginine Rich Splicing Factor 3
STAT3	Signal Transducer And Activator Of Transcription 3
SYNCRIP	SYNaptotagmin Binding Cytoplasmic RNA Binding Protein
TCA	Tricarboxylic acid cycle
TCEP	Tris(2-chloroethyl) Phosphate
TCERG1	Transcription Elongation Regulator 1
TCGA	The Cancer Genome Atlas
TEAB	Tetraethylammonium bromide
TEX10	Testis Expressed 10
TGF-β	Transforming Growth Factor Beta
TLN1	Talin 1
TNM	Tumor Node Metastasis
TNPO1	Transportin 1
TRMT112	tRNA Methyltransferase 112
TPR	Translocated Promoter Region
TPX2	Targeting Protein for Xklp2
TSR1	TSR1 Ribosome Maturation Factor
TUBA1B	Tubulin Alpha 1B
TUBA1C	Tubulin Alpha 1C
TUBB4B	Tubulin Beta 4B
TUBB	Tubulin Beta
TUFM	Tu Translation Elongation Factor, Mitochondrial
UBA52	Ubiquitin A-52 Residue Ribosomal Protein Fusion Product 1
UBC	Ubiquitin C
UBB	Ubiquitin B
UQCRC1	Ubiquinol-Cytochrome c Reductase Core Protein I
UQCRC2	Ubiquinol-Cytochrome c Reductase Core Protein II
VCP	Valosin Containing Protein
VDAC1	Voltage Dependent Anion Channel 1
VEGF	Vascular Endothelial Growth Factor
WDR61	WD Repeat Domain 61
WNT	Wnt Signaling Molecules
