# Supplementary material for: Biological Performance and Molecular Mechanisms of Mesyl MicroRNA-Targeted Oligonucleotides in Colorectal Cancer Cells
Source: Int J Mol Sci. 2025 Dec 4;26(23):11747. doi: 10.3390/ijms262311747 (PMC12692703; doi:10.3390/ijms262311747)
Supplement: Supplementary file 1 [file ijms-26-11747-s001.zip › Supplementary materials_after review_2 round.pdf]

Supplementary materials

Biological Performance and Molecular Mechanisms of Mesyl MicroRNA-Targeted Oligonucleotides in Colorectal Cancer Cells

Svetlana K. Miroshnichenko, Olga A. Patutina, Andrey V. Markov, Maxim S. Kupryushkin, Valentin V. Vlassov and Marina A. Zenkova *

Institute of Chemical Biology and Fundamental Medicine Siberian Branch of the Russian Academy of Sciences, 630090 Novosibirsk, Russia; sveta-mira@yandex.ru (S.K.M.); patutina@1bio.ru (O.A.P.); andmrkv@gmail.com (A.V.M.); kuprummax@1bio.ru (M.S.K.); vvv@niboch.nsc.ru (V.V.V.)

* Correspondence: marzen@1bio.ru or marzen@niboch.nsc.ru; Tel.: +7-383-363-51-60

Table of consent

Figure S1. Efficiency of μ-ASO delivery to Caco-2 cells mediated by Lipofectamine 2000™ ……………..2

Figure S2. Dynamics of miR-21 downregulation in Caco-2 cells……………………………………………..2

Figure S3. Comparative proteomic patterns of Caco-2 cells upon treatment with single μ-ASO targeted to miR-21, miR-17 and miR-155 and their triple combination (Combi)…………………………………………...2

Figure S4. Venn diagram illustrating shared and unique differentially expressed proteins (DEPs) across Caco-2 cells treated with miRNA-targeted μ-oligonucleotides (μ-21, μ-17, μ-155) and control oligonucleotide μ-Scr…………………………………………...……………………………………….…...3

Figure S5. Involvement of DEPs identified in groups treated with single μ-ASOs μ-21, μ-17, μ-155 and their triple combination in the key signaling pathways including WNT, MAPK, IL, Hedgehog, NOTCH, and VEGF in Caco-2 cells. …………………………………………......................................……………..…...4

Table S1. Overlap analysis of proteins implicated in core cellular functions modulated by miRNA-targeted μ-ASOs under mono- and combination treatment with canonical signaling pathway components……………..………………………………………………….................................….……………...6

Figure S6. Protein-protein interaction (PPI) subnetwork and associated biological processes for μ-21 and μ-17 treated Caco-2 cells..…………….……………………………….................................……….…….……...7

Figure S7. Protein-protein interaction (PPI) subnetwork and associated biological processes for μ-155 treated Caco-2 cells……...……………….……………………………….................................………………....8

Figure S8. Cancer-associated modules and associated biological processes for Combi treated Caco-2 cells…….....9

Table S2. Potential therapeutic and compensatory roles of top 50 DEPs upon miR-21, miR-17 and miR-155 inhibition in Caco-2 cells……………………………………………………………………………………..10

Figure S9. The effects of simultaneous application of μ-ASOs and cycloheximide in Caco-2 cells. ………………………………………………………………………………………………………..…..… ..12

Figure S10. Clinical significance of proteins altered in response to miR-21, miR-17 and miR-155 inhibition in colorectal cancer. ………………………………………………………………………………………..13

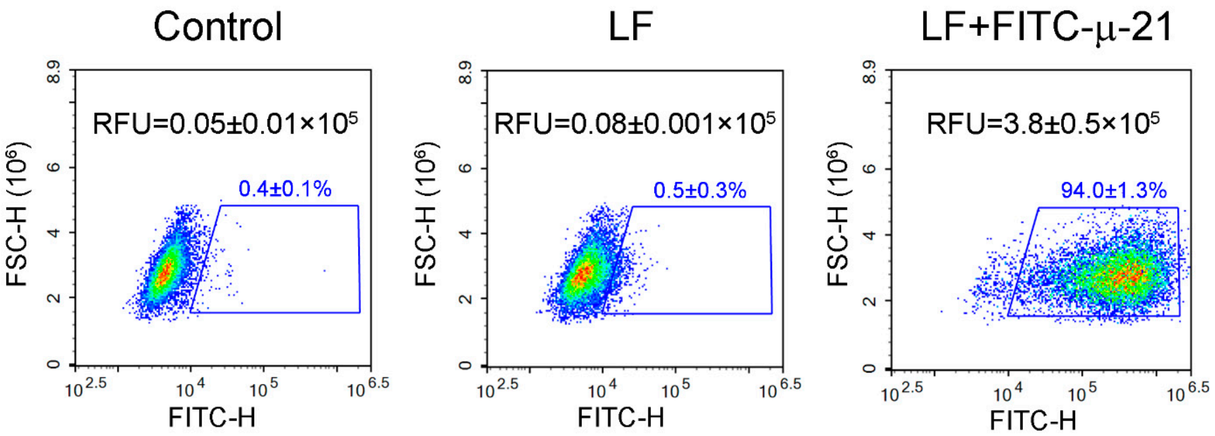

Figure S1. Efficiency of μ-ASO delivery to Caco-2 cells mediated by Lipofectamine 2000™. Results of flow-cytometry analysis 4 h post-transfection. Control – intact Caco-2 cells. LF – Caco-2 cells treated with Lipofectamine 2000™ without oligonucleotide. FITC-μ-21 – Caco-2 cells transfected with FITC-labelled μ-21 in complex with Lipofectamine 2000™ (150 nM).

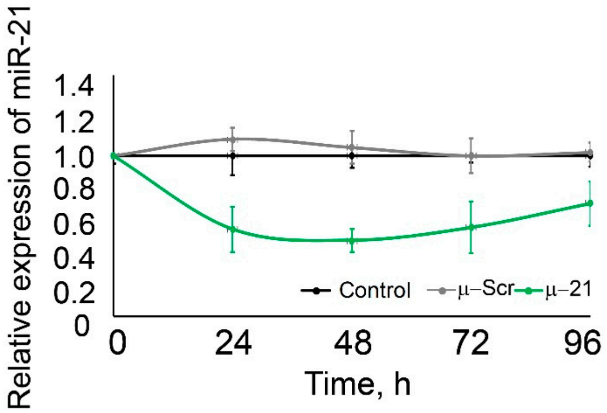

Figure S2. Dynamics of miR-21 downregulation in Caco-2 cells. miR-21 level in Caco-2 cells at 24, 48, 72, and 96 h post-transfection with a control oligonucleotide (μ-Scr) or μ-21 (120 nM) complexed with Lipofectamine 2000™. Control − intact Caco-2 cells. Detection was performed by stem-loop PCR. miR-21 level was normalized to the level of U6 snRNA.

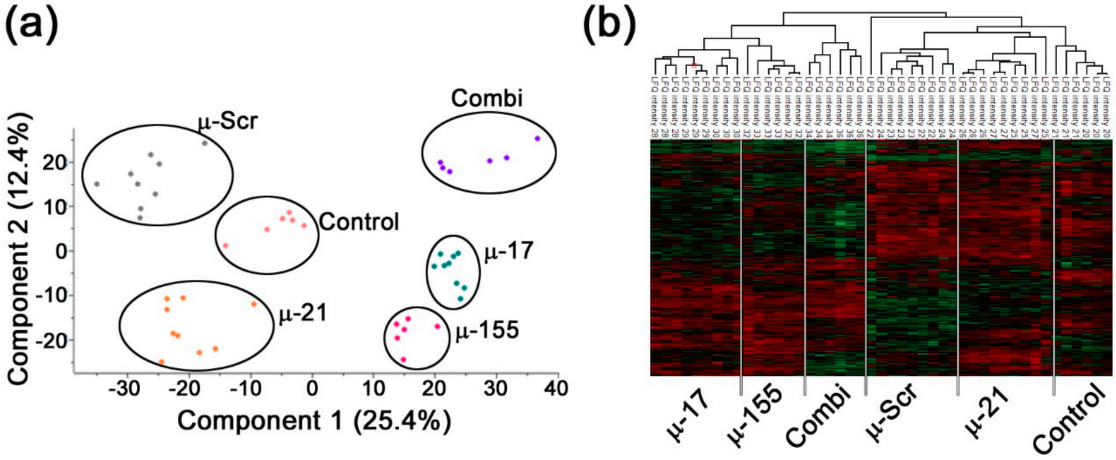

Figure S3. Comparative proteomic patterns of Caco-2 cells upon treatment with single μ-ASO targeted to miR-21, miR-17 and miR-155 and their triple combination (Combi). (**a**) Principal component analysis (PCA) demonstrating the segregation of treated groups based on protein expression patterns, exhibited more than 2-fold change in expression. (**b**) Hierarchical clustering heatmap of the protein expression profiles across experimental groups. The proteomic profiling of Caco-2 cells was carried out 72 h after transfection with control oligonucleotide (μ-Scr), miRNA-targeted μ-21, μ-17, and μ-155 or their combination Combi (μ-21+μ-17+μ-155) pre-complexed with Lipofectamine2000™ (total concentration 120 nM).

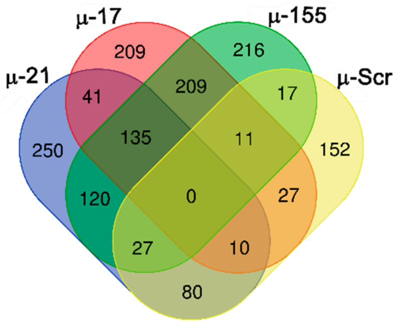

Figure S4. Venn diagram illustrating shared and unique differentially expressed proteins (DEPs) across Caco-2 cells treated with miRNA-targeted μ-oligonucleotides (μ-21, μ-17, μ-155) and control oligonucleotide μ-Scr. DEPs were defined as |log2(Fold Change (FC)| ≥1, FDR<0.05.

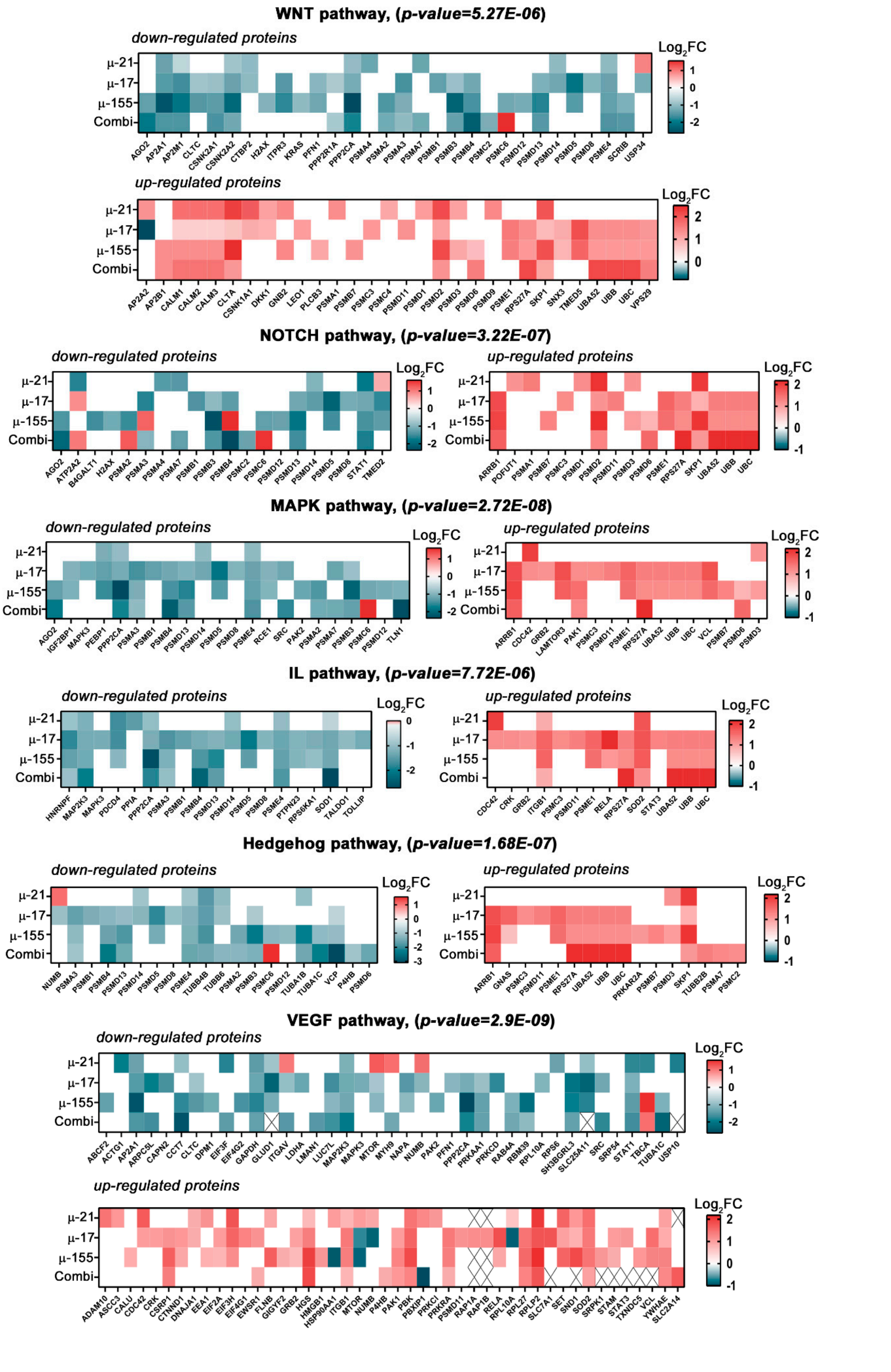

Figure S5. Involvement of DEPs identified in groups treated with single μ-ASOs μ-21, μ-17, μ-155 and their triple combination in the key signaling pathways including WNT, MAPK, IL, Hedgehog, NOTCH, and VEGF in Caco-2 cells. Analysis was carried out based on full DEPs list for each cohort. Heatmaps are

composed based on log_2FC of DEPs, involved in corresponding signaling pathway. Blue color – down-regulated protein ($log_2FC<0$), red color – up-regulated protein ($log_2FC>0$), white color – the absence of statically significant changes in protein expression, “X” – the protein was not detected in respective μ-ASO-treated cohort. Analysis was performed using ClueGo v2.5.9 plugin (Cytoscape) with annotations from Gene Ontology, KEGG, Wiki Pathways.

Table S1. Overlap analysis of proteins implicated in core cellular functions modulated by miRNA-targeted μ-ASOs under mono- and combination treatment with canonical signaling pathway components. The overlap was calculated as the percentage of proteins jointly annotated to both μ-ASO-regulated functional clusters and signaling pathways. Functional annotation was performed by ClueGo v2.5.9 plugin (Cytoscape) using annotations from Gene Ontology, KEGG, Wiki Pathways. Red color indicates the highest percentage of cross-section of proteins between signaling pathway and functional term.

ON	**VEGF**	**Hedgehog**	**WNT**	**NOTCH**	**MAPK**	**IL**	**Function**
μ-21	4.9	4.9	8.6	3.7	6.2	3.7	**Metabolism of RNA**
μ-17	3.8	0	1.3	0	1.3	2.6	
μ-155	6.2	0	1.2	1.2	2.5	1.3	
Combi	3.8	12.7	13.9	11.4	12.7	6.3	
μ-21	6.25	16.7	16.7	8.3	10.4	6.25	**Cell Cycle, Mitotic**
μ-17	10.9	30.9	30.9	25.5	32.7	30.9	
μ-155	7.6	30.3	30.3	25.8	25.8	16.7	
Combi	8	32	28	20	22	10	
μ-21	7.1	28.6	42.8	21.4	29	14.3	**Apoptosis**
μ-17	20.8	62.5	62.5	58.3	66.7	70.8	
μ-155	10	53.3	53.5	50	56.7	33.3	
Combi	5.9	58.8	41.2	52.9	52.9	23.6	
μ-21	12	0	0	0	0	1.3	**Peptide metabolic process**
μ-17	10.8	1.96	1.96	2.9	2.9	5.9	
μ-155	8.9	1.8	3.6	3.6	3.6	4.5	
Combi	7.9	0	2.6	1.3	1.3	2.6	
μ-21	11.1	0	0	0	0	0	**Translation**
μ-17	8.5	3.4	3.4	3.4	3.4	3.4	
μ-155	3.1	3.1	3.1	3.1	3.1	3.1	
Combi	9.3	0	0	0	0	0	
μ-21	8.3	25	0	0	0	0	**Macroautophagy**
μ-17	5.6	33.3	27.8	22.2	33.3	22.2	
μ-155	20	40	25	20	25	20	
Combi	17.6	35.9	11.8	0	5.9	0	
μ-21	12.7	14.5	12.7	7.3	7.3	7.3	**Cellular responses to stimuli**
μ-17	14.9	18.1	17	14.9	18.1	24.5	
μ-155	10.8	22.6	20.4	18.3	19.4	15	
Combi	12.3	25.7	18.6	14.3	14.3	10	
μ-21	0	0	0	0	0	0	**Aerobic respiration**
μ-17	0	0	0	0	0	0	
μ-155	0	3.6	0	0	0	0	
Combi	0	6.3	0	0	0	0	
μ-21	0	0	0	0	0	5.3	**RNA localization**
μ-17	0	0	0	0	4.2	4.2	
μ-155	4.2	0	0	0	4.2	0	
Combi	5.6	0	0	0	0	0	

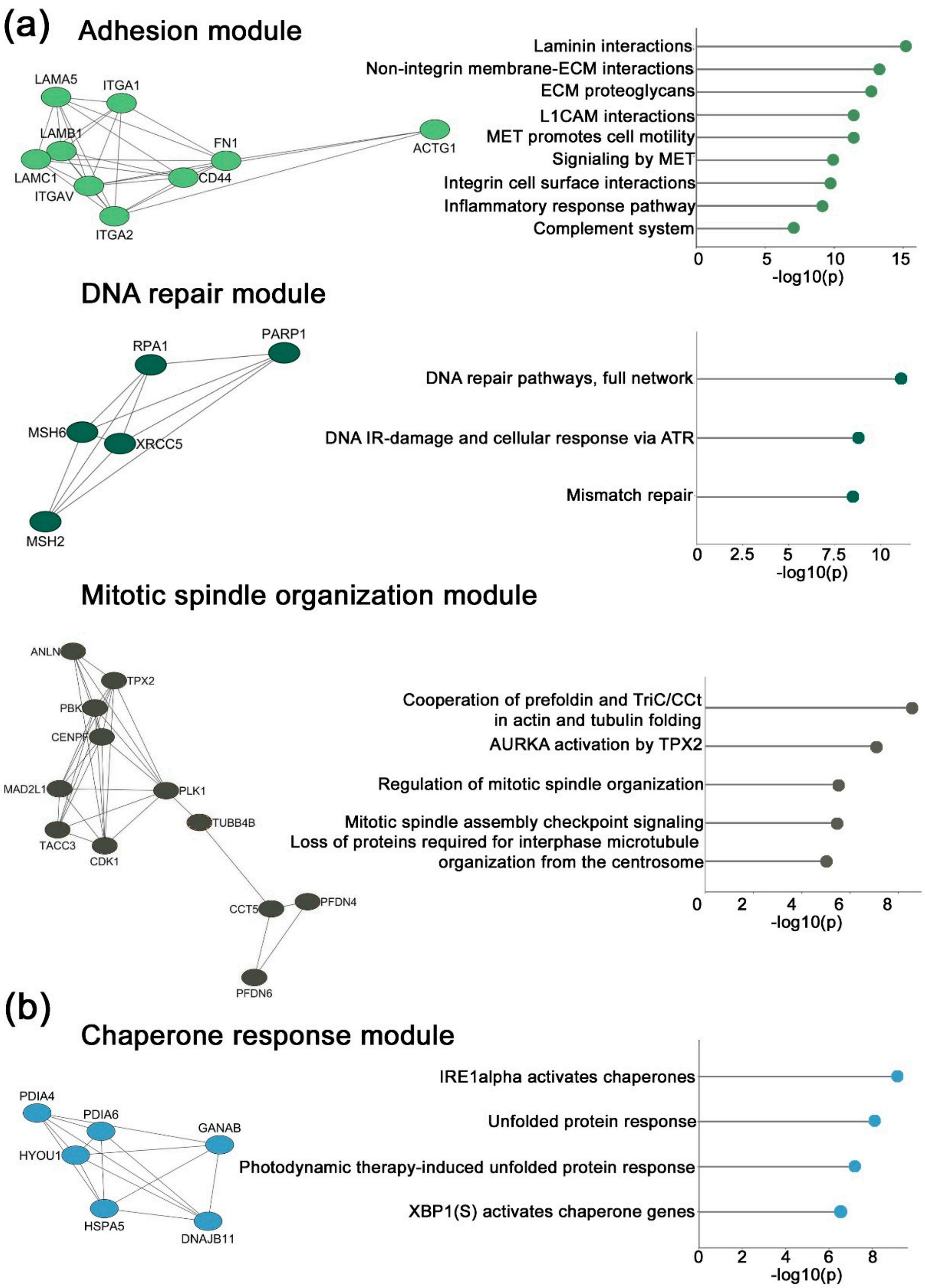

Figure S6. Protein-protein interaction (PPI) subnetwork and associated biological processes for μ-21 (**a**) and μ-17 (**b**) treated Caco-2 cells. Nodes represent DEPs (|log2FC| >1), with edges indicating high-confidence interactions (STRING score >0.7). Distinct colors represent functional modules of DEPs associated with specific biological processes in Caco-2 cells (MCODE score > 5). Cluster analysis was performed using the MCODE v2.0.3 plugin (Cytoscape), functional annotaiton was performed by ClueGo v2.5.9 plugin (Cytoscape) using annotations from Gene Ontology, KEGG, Wiki Pathways.

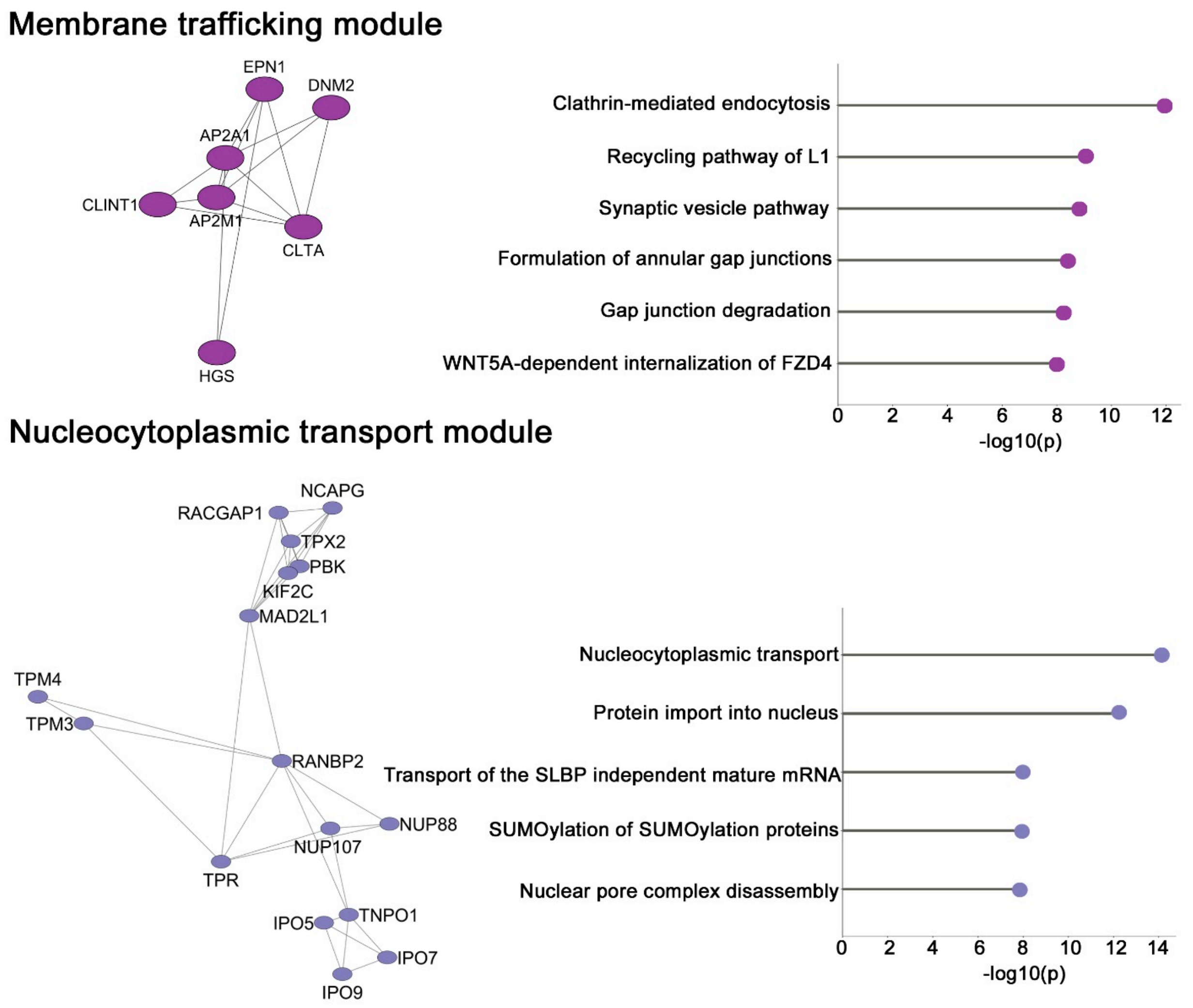

Figure S7. Protein-protein interaction (PPI) subnetwork and associated biological processes for μ-155 treated Caco-2 cells. Nodes represent DEPs (|log2FC| >1), with edges indicating high-confidence interactions (STRING score >0.7). Distinct colors represent functional modules of DEPs associated with specific biological processes in Caco-2 cells (MCODE score > 5). Cluster analysis was performed using the MCODE v2.0.3 plugin (Cytoscape), functional annotaiton was performed by ClueGo v2.5.9 plugin (Cytoscape) using annotations from Gene Ontology, KEGG, Wiki Pathways.

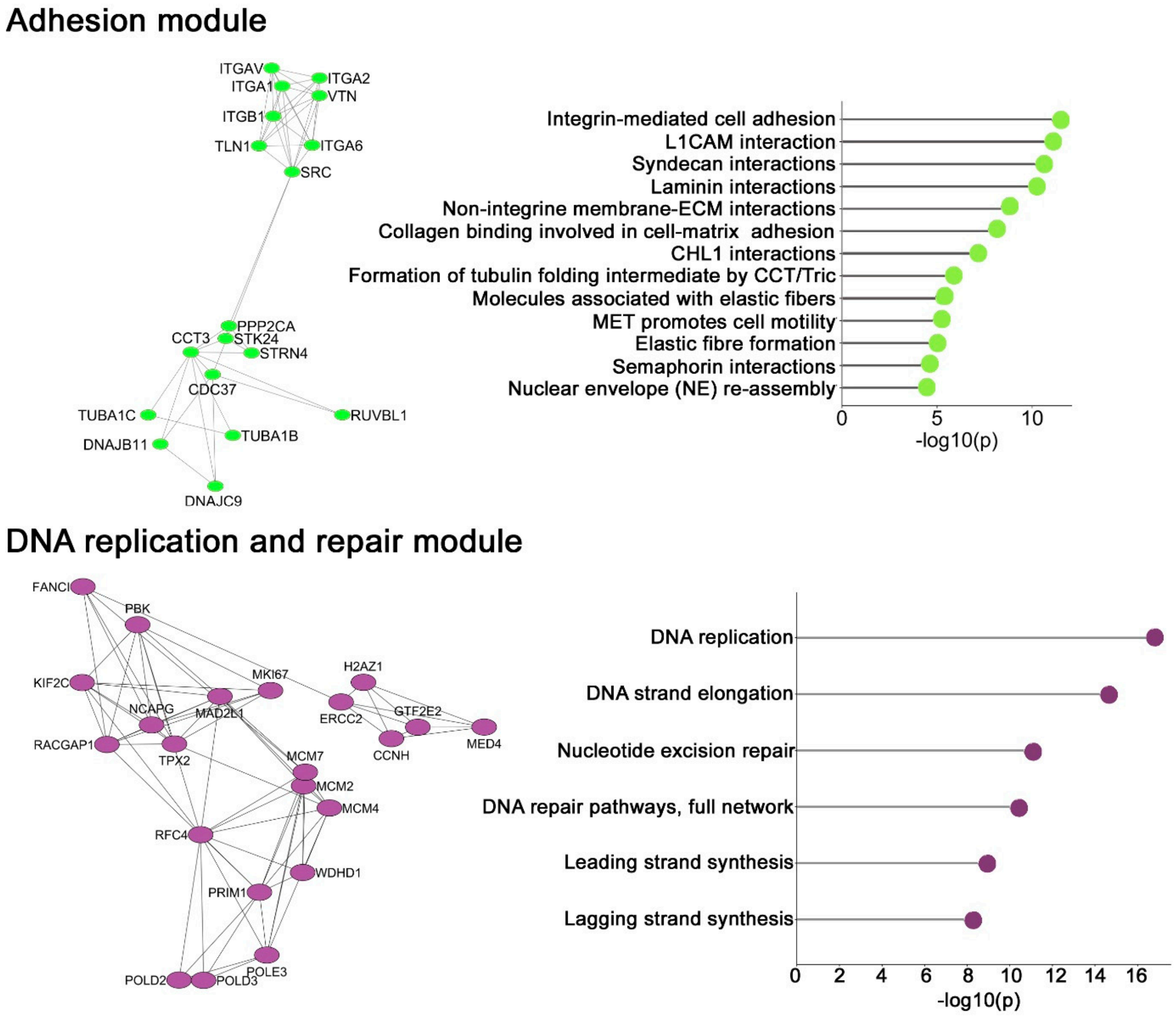

Figure S8. Cancer-associated modules and associated biological processes for Combi treated Caco-2 cells. Nodes represent DEPs (|log2FC| >1), with edges indicating high-confidence interactions (STRING score >0.7). Distinct colors represent functional modules of DEPs associated with specific biological processes in Caco-2 cells (MCODE score > 5). Cluster analysis was performed using the MCODE v2.0.3 plugin (Cytoscape), functional annotaiton was performed by ClueGo v2.5.9 plugin (Cytoscape) using annotations from Gene Ontology, KEGG, Wiki Pathways.

Table S2. Potential therapeutic and compensatory roles of top 50 DEPs upon miR-21, miR-17 and miR-155 inhibition in Caco-2 cells.

**μ-21**	
**Therapeutic effects**	**Compensatory effects**
Increased sensitivity to oxaliplatin, activation of oxidative stress response (HSPD1)<br>Reprogramming of glycolysis, lipid metabolism and mitochondrial respiration (reduced glucose consumption and ATP production) (PFKL)<br>Prevention of aneuploid cell survival and apoptosis stimulation (MAD2L1)<br>Reduction of glucose metabolism and purine biosynthesis (NDUFS3)<br>Suppression of cancer stemness, induction of caspase-3-dependent apoptosis (EXOSC10+RPA)<br>Suppression of immunosuppression (LAMB1)<br>Apoptosis stimulation through accumulation of aberrant mitoses (DENR)<br>Reprogramming of aberrant serine metabolism (eIF3F)<br>Regulation of cell cycle, apoptosis stimulation (RPLP0)	Increase in cancer stemness markers (CD44)<br>Accelerated mitotic entry and enhanced rRNA synthesis (NOL11, CDK1)<br>Upregulation of ribosomal components and translation factors (RPL37A, RPS7, RPS23)<br>Increased glucose uptake and lactate production, enhanced Warburg effect (DDX39B)<br>Suppression of apoptotic signaling (HSP90AA1)<br>Enhanced immunosuppressive microenvironment via suppression of CD8+ T-cells and NK cells (NEDD8)<br>Formation of filopodia and increased invasive potential (CDC42)<br>Upregulation of proteasomal components (PSMD3)
**μ-17**	
**Therapeutic effects**	**Compensatory effects**
Reduction of purine synthesis, suppression of cancer cell stemness, reduction of chemoresistance, proliferation and migration (ATIC)<br>Reprogramming of ammonia metabolism, reduction of glutamate levels (GLUD1, GLUD2)<br>Reduction of cancer cell stemness (ALDH)<br>Reduction of the proliferative and migratory potential of tumor cells (POLR2A, MAGOH+MAGOB)<br>Reduction of energy production (UQCRC2, UQCRC1)<br>Reduction of fatty acid beta-oxidation (ACAA1)<br>Reduction of chemoresistance, proliferation and migration, induction of apoptosis (SKP2)<br>Reduction of ER-to-Golgi transport (SEC23A)	Increased production of ribosomal components and translation initiation factors (RPS2, RPL27, RPS23, RPS4X)<br>Downregulation of proteasome assembly inhibitors (PSMD5)
**μ-155**	
**Therapeutic effects**	**Compensatory effects**
Induction of apoptosis, reduction of chemoresistance (TUB1B)<br>Stimulation of apoptosis, increased mitochondrial membrane permeability (NDAC1, TCERG1)<br>Positive regulation of nucleolar stress response (GRWD11, RPL23)<br>Suppression of aerobic glycolysis, cuproptosis (GAPDH)	Enhancement of metastatic potential (CDH1)<br>Stimulation of EMT through actin cytoskeleton regulation (ARPC2)<br>Promotion of angiogenesis (EPN1)<br>Increase in cancer cell stemness (SKP1)<br>Upregulation of transport factors (NUP107)<br>Production of proteasomal components (PSMD2)

Reduction of ATP production, increased reactive oxygen species levels (OGDH)<br>Suppression of lipogenesis and increased reactive oxygen species levels (SF3B3)<br>Normalized reprogramming of asparagine synthesis and arginine consumption (RBM39)<br>Reduction of chemoresistance (DAPS)<br>Suppression of pro-inflammatory cytokine expression (TUBA1B)<br>Inhibition of proteasomal component biogenesis (PSMB3)<br>Enhanced sensitivity to erastin-induced ferroptosis (PPP2CA)	
**Combi**	
**Therapeutic effects**	**Compensatory effects**
Enhanced sensitivity to erastin-induced ferroptosis (HSPA5)<br>Downregulation of proteasomal components (PSMD4)<br>Reduced proliferation, migration, and induction of apoptosis through decreased lipid accumulation, suppression of fatty acid synthesis, and inhibition of MYC signaling pathway (NIFK)<br>Reduction of metastatic potential (TLN1)<br>Suppression of proliferation, migration, and induction of apoptosis via caspase-3/7 activation and regulation of methyltransferase activity (SYNCRIP)<br>Reduced chromatin accessibility and remodeling (HMGN1)<br>Inhibition of protein proteolysis, including p53 degradation (BTF3, DCAF13)<br>G2/M cell cycle arrest (SNRPN)<br>Suppression of fatty acid oxidation via regulation of glutamine metabolism and reduced ATP synthesis (VCP)<br>Decreased proliferation and migration rates through inhibition of spindle assembly and protein misfolding (CCT3, CCT7)<br>Reduction of ribosome biogenesis and telomerase activity (TSR1)<br>Decreased ATP synthesis (GFM1)<br>Increased reactive oxygen species levels (SOD1)	Enhanced protein proteolysis due to the hyperexpression of ubiquitin system components (UBB, UBA52, UBC)<br>Increased ATP production and reduced generation of reactive oxygen species (ROS) (NDUFS8)<br>Stabilization of oncogene mRNAs (SRSF3)<br>Synthesis of ribosomal proteins and aberrant expression of translation initiation factors, including those responsible for the assembly of the pre-initiation complex and control of translation fidelity (RPS27A, RPS16, RPS13, RPS15, EIF1, RPS23, RPL23A)

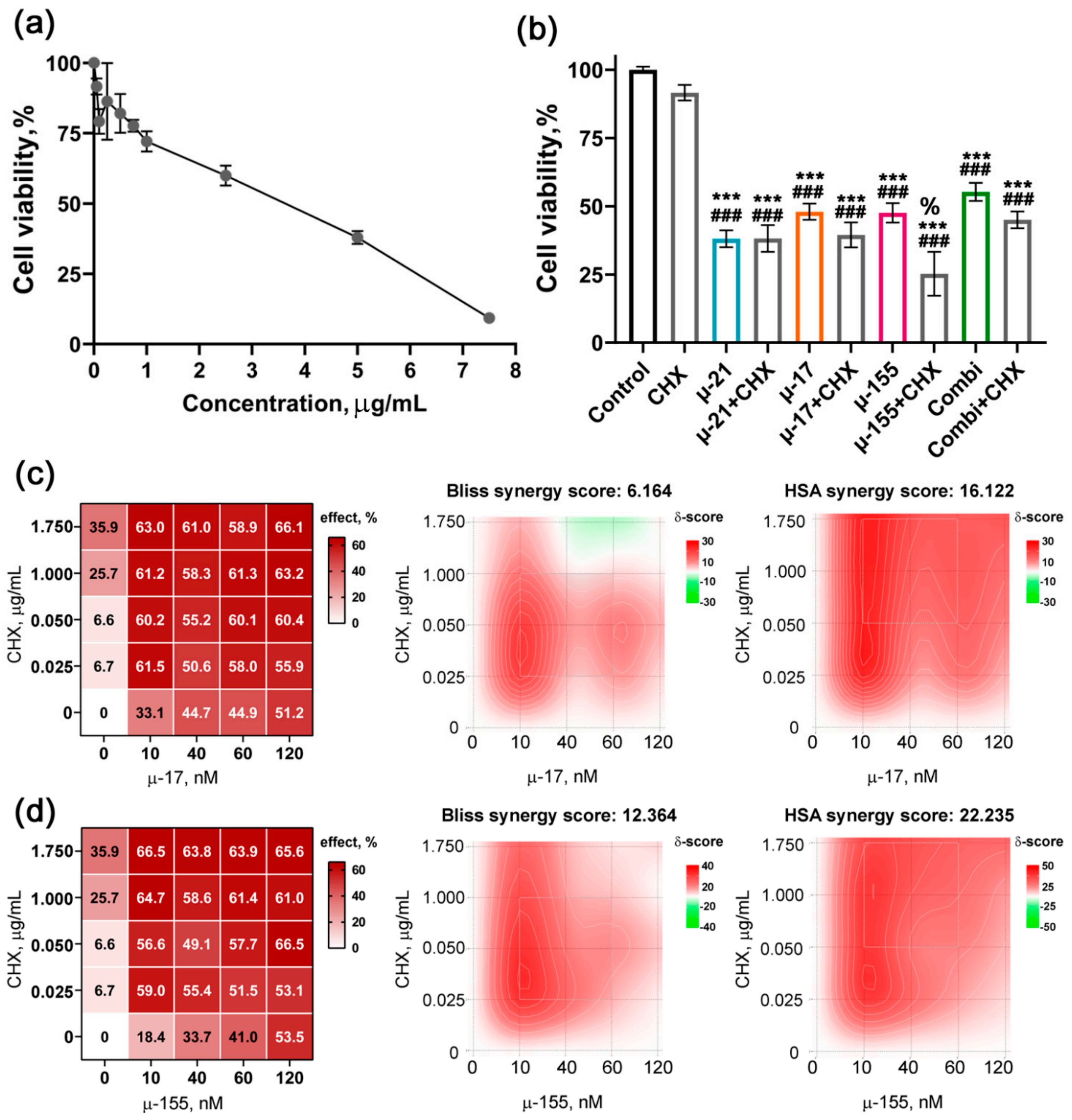

Figure S9. Effects of simultaneous application of μ-ASOs and cycloheximide (CHX) in Caco-2 cells. (**a**) Caco-2 cell viability depending on the concentration of CHX used. Results of MTT test carried out after 24 h of incubation with CHX at a concentration range 0.05-7.5 μg/mL. (**b**) The effects of concurrent treatment with μ-ASOs (total concentration 120 nM) and CHX (0.05 μg/mL). CHX was added 24 h post-transfection with μ-ASOs, MTT test was carried out 72 h post-transfection (48 h post incubation with CHX). Control – cells without treatment, CHX – cells incubated with CHX (0.05 μg/mL), μ-21, μ-17 and μ-155 – cells transfected with miRNA-targeted μ-ASOs (120 nM), Combi – cells transfected with triple combination of μ-ASOs (40 nM each, 120 nM total). μ-21+CHX, μ-17+CHX, μ-155+CHX, Combi + CHX – cells co-treated with μ-ASOs and CHX. Transfection was performed using Lipofectamine2000™. Significance was determined by one-way ANOVA with post-hoc Tukey test. ###, *** - statistically significant difference from Control and CHX with p-value<0.001, % - statistically significant difference from μ-155 with p-value<0.05. (**c**) and (**d**) Matrices demonstrating inhibition of Caco-2 cell viability by different concentrations of CHX and μ-17 (**c**) or μ-155 (**d**) applied alone or in combinations (left panels). Heatmaps (right panels) show the areas with the highest additive-synergetic effects of CHX and μ-ASOs on Caco-2 cell viability according to Bliss and HSA model. The synergy score < −10 denotes antagonism; −10–10—additive effect; and >10—synergy.

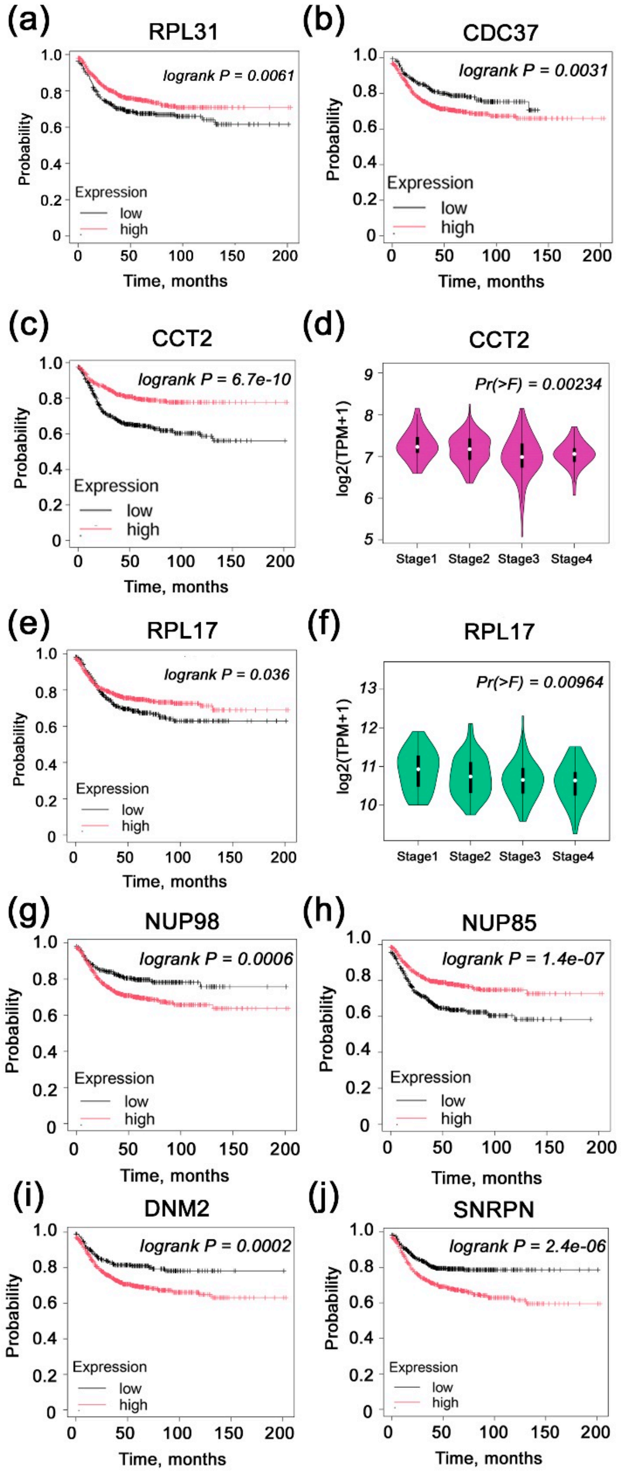

Figure S10. Clinical significance of proteins altered in response to miR-21, miR-17 and miR-155 inhibition in colorectal cancer. (**a**)-(**c**), (**e**), (**g**)-(**j**) Correlation between the expression of target proteins (from the list of top 50 differentially expressed proteins or cancer-associated modules) and relapse-free survival in colorectal cancer patients (TCGA dataset). Patients with high and low expression levels of corresponding mRNAs are indicated in red and black, respectively. (**d**), (**f**) Association of CCT2 (**d**) and RPL17 (**f**) expression level with tumor stage (I–IV) in colorectal adenocarcinoma patients. Analysis was performed using Gepia2. The presented target proteins possess a predicted binding site for corresponding miRNA: RPL31 for miR-21, RPL17 for miR-17 and miR-155, DNM2 and SNRPN for miR-17, or were previously validated as direct targets of these miRNAs by established methods (PAR-CLIP, dual luciferase assay) (CCT2 and CDC37 – miR-21, NUP98 and NUP85– miR-17).
